# Systematic functional profiling of transcription factor networks in *Cryptococcus neoformans*

**DOI:** 10.1038/ncomms7757

**Published:** 2015-04-07

**Authors:** Kwang-Woo Jung, Dong-Hoon Yang, Shinae Maeng, Kyung-Tae Lee, Yee-Seul So, Joohyeon Hong, Jaeyoung Choi, Hyo-Jeong Byun, Hyelim Kim, Soohyun Bang, Min-Hee Song, Jang-Won Lee, Min Su Kim, Seo-Young Kim, Je-Hyun Ji, Goun Park, Hyojeong Kwon, Suyeon Cha, Gena Lee Meyers, Li Li Wang, Jooyoung Jang, Guilhem Janbon, Gloria Adedoyin, Taeyup Kim, Anna K. Averette, Joseph Heitman, Eunji Cheong, Yong-Hwan Lee, Yin-Won Lee, Yong-Sun Bahn

**Affiliations:** 1 Department of Biotechnology, Center for Fungal Pathogenesis, College of Life Science and Biotechnology, Yonsei University, Seoul 120-749, Korea; 2 Department of Biotechnology, College of Life Science and Biotechnology, Yonsei University, Seoul 120-749, Korea; 3 Department of Agricultural Biotechnology, Center for Fungal Pathogenesis, Seoul National University, Seoul 151-921, Korea; 4 Unité Biologie et Pathogénicité Fongiques, Département de Mycologie, Institut Pasteur, Paris F-75015, France; 5 Department of Molecular Genetics and Microbiology, Medicine, and Pharmacology and Cancer Biology, Duke University Medical Center, Durham, North Carolina 27710, USA

## Abstract

*Cryptococcus neoformans* causes life-threatening meningoencephalitis in humans, but its overall biological and pathogenic regulatory circuits remain elusive, particularly due to the presence of an evolutionarily divergent set of transcription factors (TFs). Here, we report the construction of a high-quality library of 322 signature-tagged gene-deletion strains for 155 putative TF genes previously predicted using the DNA-binding domain TF database, and examine their *in vitro* and *in vivo* phenotypic traits under 32 distinct growth conditions. At least one phenotypic trait is exhibited by 145 out of 155 TF mutants (93%) and ∼85% of them (132/155) are functionally characterized for the first time in this study. The genotypic and phenotypic data for each TF are available in the *C. neoformans* TF phenome database (http://tf.cryptococcus.org). In conclusion, our phenome-based functional analysis of the *C. neoformans* TF mutant library provides key insights into transcriptional networks of basidiomycetous fungi and human fungal pathogens.

## Abstract (web summary)

*Cryptococcus neoformans* is a fungal pathogen that causes meningoencephalitis. Here the authors describe the production of a gene-deletion mutant collection representing most *C. neoformans* non-essential transcription factors, providing insight into the signalling networks that govern the biology and pathogenicity of this fungus.

## Graphical abstract

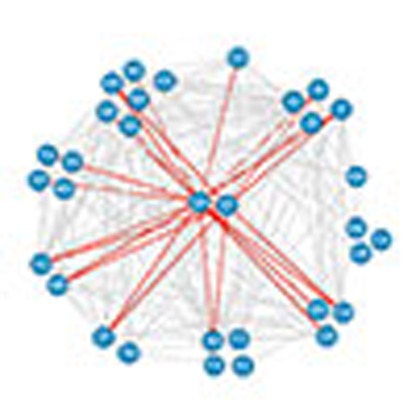

C*ryptococcus neoformans* is a basidiomycete fungal pathogen that causes meningoencephalitis—mainly in immunocompromised populations—and is responsible for more than 600,000 deaths annually worldwide^1^. However, limited therapeutic options are available for treating cryptococcosis^2^, and a complete understanding of diverse biological aspects of *Cryptococcus* is urgently required for developing novel therapeutic targets and methods. To this end, the signalling cascades governing the general biology and pathogenicity of *C. neoformans* have been extensively studied over the past decades. This has allowed us to understand several key metabolic and signalling pathways in this pathogen, including those involving Ras, cAMP, Rim101, calcineurin, three MAPKs (Cpk1, Mpk1 and Hog1) and the unfolded protein response (UPR)^3^.

Most of the aforementioned signalling cascades are composed of sensor and receptor-like proteins and kinases or phosphatases, and are often equipped with unique adaptor or scaffolding proteins to enhance the specificity of each signalling pathway to prevent aberrant crosstalk. Nevertheless, each signalling cascade ultimately activates or represses its effector proteins through transcription factor (TF) binding to specific promoters to regulate transcription. Repertoires of TFs are often more divergent among species than are those of other signalling components. This appears particularly true in the case of *C. neoformans*, as evident from recent genome analyses^4^. Therefore, *C. neoformans* appears to possess numerous evolutionarily conserved signalling cascades featuring divergent sets of TFs, which might govern the characteristics of *C. neoformans* that are unique compared with those of other fungi.

To understand *C. neoformans* TF networks on a global scale, we constructed a high-quality gene-deletion collection through homologous recombination methods for 155 putative *C. neoformans* TFs previously predicted to contain DNA-binding domains (DBDs)^5^,^6^. The TF mutant strains are analysed for 30 distinct *in vitro* phenotypic traits, which cover growth, differentiation, stress responses, antifungal resistance and virulence-factor production. Moreover, we also performed a large-scale virulence test using an insect host model and signature-tagged mutagenesis (STM) scoring in a murine host model. This comprehensive phenotypic data set (phenome) of the TFs, which can be accessed online through the *Cryptococcus* Transcription Factor Phenome Database (http://tf.cryptococcus.org) provides a unique opportunity to understand general biological features of *C. neoformans* and identifies novel putative pathways that could be targeted for the treatment of cryptococcosis. This TF mutant collection and its phenome data are a valuable resource for those studying *Cryptococcus* and the general fungal research community.

## Results

### *Cryptococcus* transcription factor mutant collection

**Figure 1:**
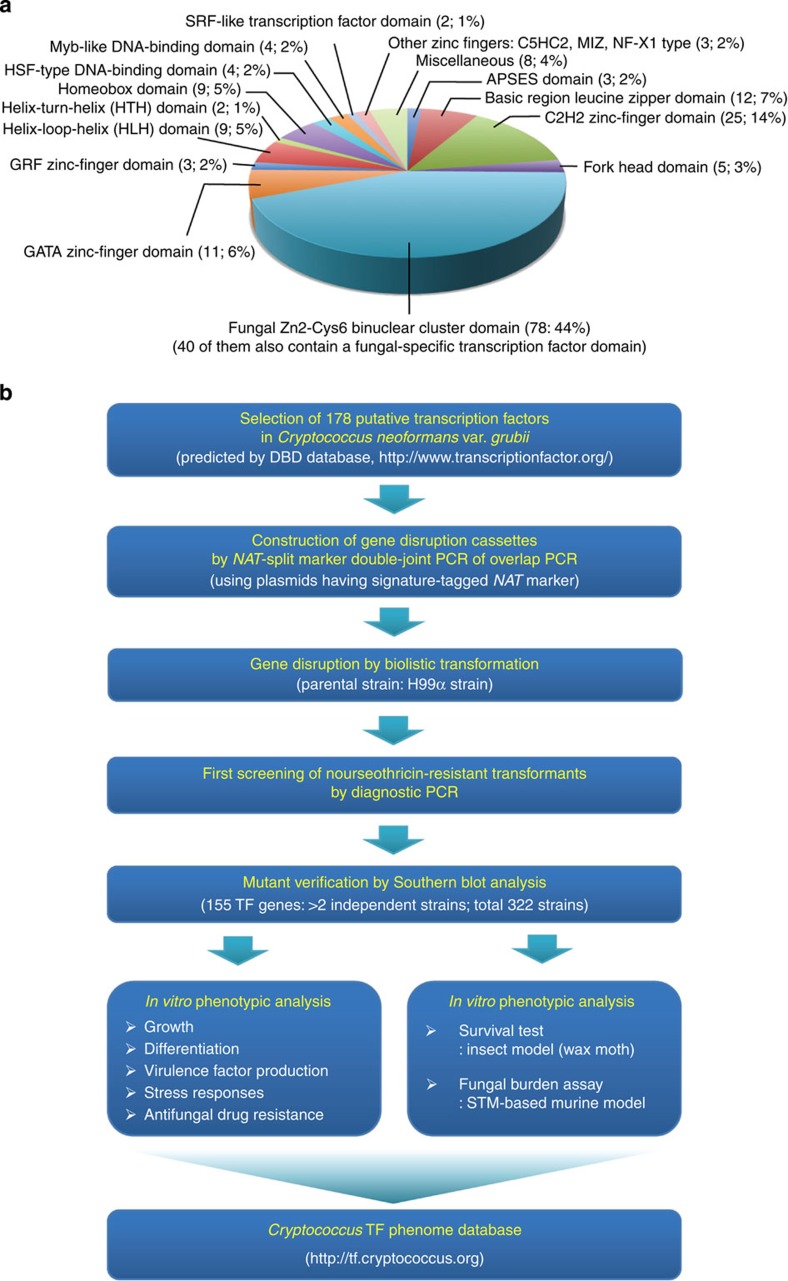
Overview of *Cryptococcus neoformans* transcription factors and strategies for their systematic deletion and phenome-based analysis. (**a**) Pie chart showing the class and distribution of *C. neoformans* TFs. Each TF was classified based on the DBDs predicted using Superfamily (http://www.supfam.org/SUPERFAMILY/) and Pfam (http://pfam.xfam.org/) databases or *Cryptococcus* genome database (http://www.broadinstitute.org). Certain TFs contain multiple DBDs (Supplementary Data 1). (**b**) Flowchart of the construction of the *C. neoformans* TF mutant library and *in vitro* and *in vivo* phoneme-based analyses.

We first selected putative TFs using the published DBD TF prediction database (http://www.transcriptionfactor.org/)^6^. The *C. neoformans* H99 strain, a serotype A platform strain, contains 188 TFs (148 predicted from Pfam and 96 from SUPERFAMILY). Because these TFs were predicted based on the first version of the annotated H99 genome database, we updated this database with reference to the most recent version (ver.7) of the annotated H99 genome database^4^, which resulted in a final prediction of 178 TFs (Supplementary Data 1). Orthologue mapping based on the BLAST *e-*value matrix demonstrated that *C. neoformans* contains several evolutionarily distinct groups of TFs (Supplementary Data 3). The *Cryptococcus* DBD TFs were classified based on their DBDs (Fig. 1a). Nearly 44% of these TFs (78) contain a fungal Zn2-Cys6 DBD, and among these, 40 also harbour a fungal-specific TF domain. Several TFs contain more than two TF domains (Supplementary Data 1).

To analyse the functions of the TFs, we deleted 155 putative TF genes out of 178 using homologous recombination. To perform a large-scale virulence test, dominant nourseothricin-resistance markers (*NATs*) containing a series of signature tags of distinct oligonucleotide sequences were employed (Supplementary Data 2). The genotypes of all TF mutants were confirmed by performing Southern blot analysis to verify both the gene deletion and the absence of any ectopic integration of each gene-disruption cassette. To accurately validate the phenotype and exclude unlinked mutational effects, we generated more than two independent TF mutants for all 155 TFs, including 4 TFs (*HXL1*, *ATF1*, *MBS1* and *SKN7*) that we previously reported^7^,^8^,^9^, and thus obtained a total of 322 strains. For parallel *in vitro* and *in vivo* phenotypic analysis, we deleted 53 TF genes, which were previously deleted in the CMO18 strain (a less virulent H99 strain)^10^, and derived more than two independent mutants. Certain known TFs, including *RIM101*, *ADA2*, *CUF1*, *SXI1*, *SP-1/CRZ1*, *NRG1*, *STE12*, *BWC2*, *SRE1*, *ZNF2* and *HAP1/HAP2*, were also independently deleted here to accurately compare phenotypes. When two independent TF mutants showed inconsistent phenotypes because of inter-isolate inconsistency, additional TF mutants were generated to exclude outlier mutants. We found that about 8% of gene knockouts (13 TFs) exhibited inconsistent phenotypes, potentially attributable to undetectable mutational artefacts or unexpected alterations in the genome (Supplementary Data 4). This level is highly similar to that reported in a similar study on the ascomycete fungal pathogen *Candida albicans*^11^. For the remaining 23 TFs, we could not generate TF mutants. Among these TFs, 6 (*ESA1*, *CEF1*, *CDC39*, *RSC8*, *HSF1* and *PZF1*) are orthologous to yeast TFs that are essential for growth of *Saccharomyces cerevisiae*. The remaining 17 TF genes could not be deleted even after repeated attempts at gene disruption and thus they are presumed to be critical or essential for growth in *C. neoformans*. However, *CIR1*, *MIG1*, CNAG_06252 and CNAG_04798 have been successfully deleted previously^10^,^12^,^13^, suggesting that these TFs could be deleted through additional efforts in the future. In summary, we successfully constructed a *C. neoformans* TF mutant collection that covers 155 TFs and 322 TF mutant strains in total (Fig. 1b).

Out of the 155 TFs whose mutants were constructed, 57 TF genes possess names designated in published studies or reserved by other researchers through registration in FungiDB (www.fungidb.org). For the remaining 98 TFs, we provided gene names by following the systematic genetic nomenclature flowchart in *C. neoformans* recently reported by Inglis *et al.*^14^ (Supplementary Data 1).

### Phenotypic profiling of the *Cryptococcus* TF mutant library

**Figure 2:**
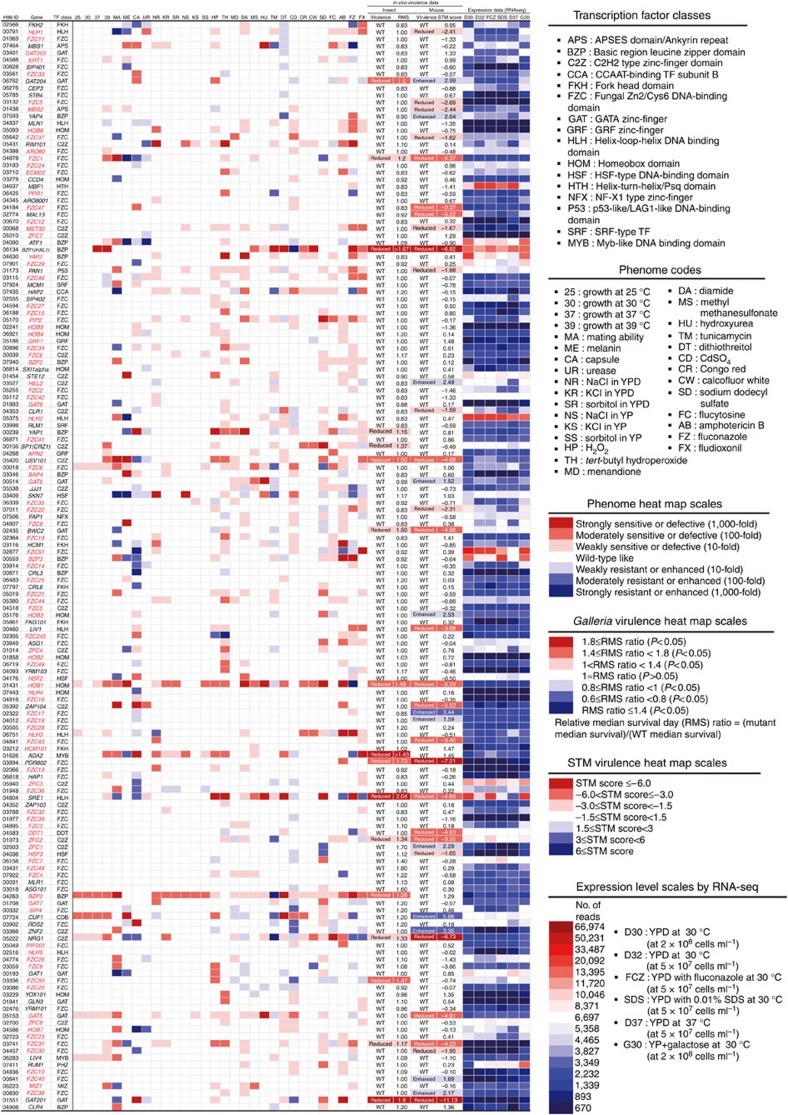
Phenome heat map created for *C. neoformans* TF mutant strains. Phenotype scores are represented in distinct colours based on qualitative or semi-quantitative measurement of >2 independent TF mutant strains under indicated growth conditions (abbreviated in the right panel). Red and blue in the heat map represent reduction and enhancement, respectively. Phenotype strengths (strong, intermediate and weak) are distinguished in gradients of red or blue, as indicated in the right panel. Genes reported previously or genes whose names are reserved in FungiDB^14^ are denoted in black, whereas genes that we newly named in this study are denoted in red. Transcriptional levels for each TF under distinct conditions were retrieved from the RNA sequencing data, which were reported in the previous *C. neoformans* H99 genome paper^4^.

For the 322 TF mutants constructed, we performed a series of phenotypic analyses for the following phenotypic classes: growth, differentiation and morphology, stress responses, antifungal drug resistance, virulence-factor production and *in vivo* virulence (Fig. 1b). This overall phenome data set generated for the TF mutant collection is illustrated together with a colour scale in the phenome heat map in Fig. 2 and Supplementary Data 4. Data for transcript levels of each TF measured by RNA sequencing analyses under six distinct growth conditions were obtained from a recent H99 genome analysis report^4^ and also demonstrated as a heat map (Fig. 2; Supplementary Data 4). The phenotypic analysis revealed that about 93% of the TF mutants (145/155) exhibited at least one discernable phenotype, suggesting a high functional coverage of this TF mutant collection. Almost 85% of the TFs (132/155) have not been functionally characterized before in *C. neoformans.* All of these phenome data are publicly available in the *Cryptococcus neoformans* TF database (http://tf.cryptococcus.org).

### TFs governing growth and differentiation of *C. neoformans*

*C. neoformans* undergoes both saprobic and pathogenic life cycles in natural and animal host environments. Therefore, it must be capable of growing at temperatures ranging from ambient (25 °C) to high (37–39 °C). Deletion of some TFs (*BZP2*, *CUF1*, *LIV4*, *GAT5*, *FZC6* and *NRG1*) resulted in temperature-independent growth defects (Supplementary Fig. 1). The growth defect of the *cuf1*Δ mutant was due to its inability to uptake copper, because external addition of CuSO_4_ restored its wild-type (WT) growth (Supplementary Fig. 2). Deletion of the following group of TFs caused growth defects only at high temperature (37–39 °C): *HXL1*, *CRZ1*, *ATF1*, *ADA2*, *HAP1*, *ARO80*, *USV101*, *FZC31*, *FZC30, FZC1*, *MIZ1*, *APN2*, *GAT6*, *MBS2*, *SRE1* and *ERT1* (Supplementary Fig. 1). Among these, only *HXL1*, which is a TF downstream of the Ire1 kinase in the UPR pathway^9^, exhibited a severe growth defect at host physiological temperature. The *hob1*Δ and *hlh3*Δ mutants showed WT growth at 30 °C but exhibited growth defects at 25 °C or high temperature. By contrast, deletion of *MLN1*, *MCM1* and *FZC46* promoted the growth of *C. neoformans* at 39 °C. Collectively, these results suggest that multiple TFs (total 27 TFs) control—both positively and negatively—the growth and thermotolerance of *C. neoformans*.

**Figure 3:**
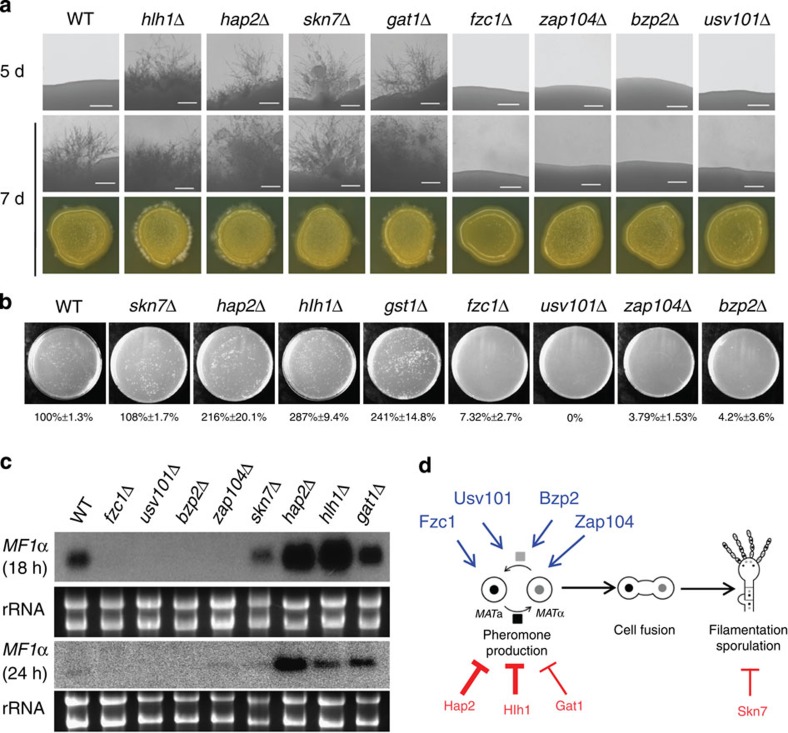
TFs involved in sexual differentiation in *C. neoformans*. (**a**) TFs required for sexual differentiation of *C. neoformans*. In the mating assay, the WT strain H99 and each TF mutant were cocultured with the opposite mating type KN99**a** strain on V8 media and incubated at room temperature in the dark for 7 days. Cells and the periphery of colonies were visualized under a microscope (upper two panels: × 100 magnification; bottom panel: × 7.5 magnification) equipped with a digital camera. Scale bars, 200 μm. (**b**) TFs involved in cell fusion. Cell-fusion efficiency of each TF mutant was calculated relative to that of control strains (*NAT-*marked WT α strain (YSB119) × *NEO-*marked WT **a** strain (YSB121)). (**c**) TFs involved in pheromone gene expression. Indicated TF mutants were cocultured with the KN99**a** strain on V8 medium at room temperature for 18 or 24 h and then cells were collected for total RNA isolation. (**d**) The proposed model for the role of Fzc1, Usv101, Bzp2, Zap104, Hap2, Hlh1, Gat1 and Skn7 in various mating stages of *C. neoformans*.

In a natural environment, *C. neoformans* exists mainly in the yeast form but undergoes either bisexual differentiation with cells of the opposite mating type or unisexual differentiation with cells of the same mating type to produce filamentous forms and generate infectious basidiospores. These developmental processes contribute to the generation of the genetic diversity of the pathogen^15^. Our systematic analysis revealed that 37 TFs were involved in mating (Fig. 3; Supplementary Fig. 3). Among the novel mating-regulating TFs discovered in this study, deletion of *BZP2*, *USV101*, *FZC1* and *ZAP104* severely reduced mating, even in unilateral matings, whereas deletion of *HLH1*, *HAP2* and *GAT1* highly enhanced mating efficiency (Fig. 3a). To determine the mating steps in which these TFs are involved, we measured the efficiency of cell fusion and pheromone production, which precede the filamentation step. The *bzp2*Δ, *usv101*Δ, *fzc1*Δ and *zap104*Δ mutants lacked the ability to engage in cell fusion with the *MAT***a** control strain (Fig. 3b) and also failed to induce pheromone gene (*MF*α*1*) expression upon mating (Fig. 3c), suggesting that Bzp2, Usv101, Fzc1 and Zap104 promote pheromone gene expression, which results in a subsequent increase in cell fusion. Conversely, in the *hlh1*Δ, *hap2*Δ and *gat1*Δ mutants, cell-fusion efficiency was increased two- to threefold (Fig. 3b) and pheromone gene expression was highly enhanced (Fig. 3c), suggesting that Hlh1, Hap2 and Gat1 are negative regulators of pheromone gene expression. By contrast, *SKN7*, whose deletion promoted mating (Fig. 3a), was dispensable for both pheromone gene expression and cell fusion (Fig. 3b,c), indicating that it is likely involved in a later stage of mating. In summary, numerous TFs are involved in the different mating steps of *C. neoformans*, demonstrating that multiple signalling cascades modulate its development.

### TFs modulating virulence-factor production in *C. neoformans*

To support survival and proliferation within the host, *C. neoformans* is armed with several virulence factors, which include capsule and melanin. Capsule is a glucuronoxylomannan- or galactoxylomannan-based polysaccharide that protects cells from being phagocytosed by host phagocytic cells^16^. Melanin, a black-brown pigment made of polyphenol complexes, confers both antiphagocytic and antioxidant activity to cells^17^.

**Figure 4:**
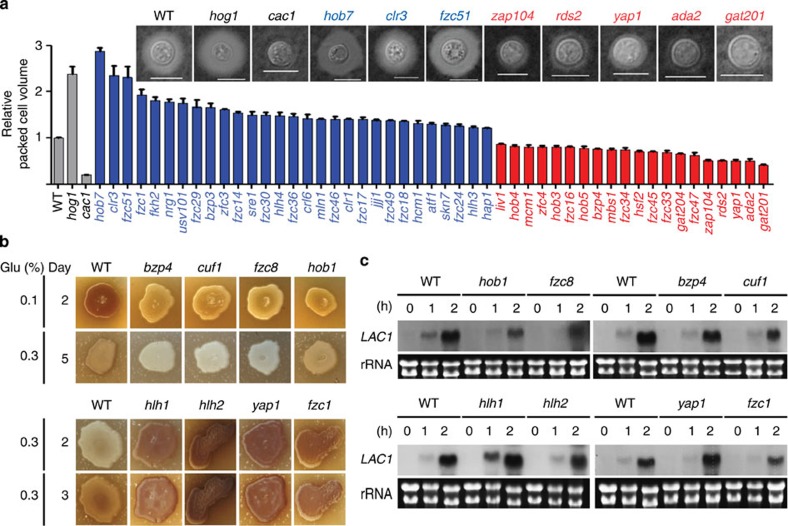
TFs involved in virulence-factor production in *C. neoformans*. (**a**) TFs involved in capsule production. WT strain H99 and TF mutants were spotted onto a Dulbecco's Modified Eagle's (DME) agar medium and incubated at 37 °C for 2 days. Scraped cells were resuspended in distilled water and visualized by means of India ink staining (see inserted pictures in the graph; scale bars, 10 μm). Each graph indicates average relative packed cell volume of two independent TF mutants that exhibited statistically significant changes in capsule production (*P<*0.05) as shown in Supplementary Fig. 4. Triplicate technical experiments with two or more independent strains were performed. Statistical difference in relative packed cell volume was determined by one-way analysis of variance with Bonferroni’s multiple-comparison test. Error bars indicate s.e.m. The *hog1*Δ and *cac1*Δ mutants were used as capsule-enhanced and capsule-defective control strains, respectively. (**b**,**c**) TFs involved in melanin production. (**b**) Indicated TF strains were spotted and grown on Niger seed agar medium (with 0.1 and 0.3% glucose) at 37 °C. The plates were photographed daily. (**c**) Northern blot analyses were performed using total RNA isolated from cells under glucose-rich (0 h) and -depleted conditions (1 and 2 h). Each membrane was hybridized with a *LAC1*-specific probe, washed and developed.

Our systematic analysis identified 49 TFs (20 positive and 29 negative regulators) involved in capsule production (Fig. 4a; Supplementary Fig. 4). In addition to previously reported capsule-regulating TFs, such as Ada2 (ref. 18), Gat201 (ref. 10), Atf1 (ref. 8) and Mbs1 (ref. 7), we identified several novel capsule-regulating TFs in this study. The *zap104*Δ, *yap1*Δ and *rds2*Δ mutants also exhibited severely reduced capsule production, on par with the extent observed in the *gat201*Δ and *ada2*Δ mutants (Fig. 4a). By contrast, deletion of *HOB7*, *CLR3* and *FZC51* greatly enhanced capsule production (Fig. 4a).

Our analysis also uncovered 27 TFs (11 positive regulators and 16 negative regulators) involved in melanin production (Fig. 4b; Supplementary Fig. 5). A few of these TF, including Cuf1, Ste12, Mbs1, Skn7 and Atf1, have been reported previously^7^,^8^,^19^,^20^,^21^. In addition, we found that the *fzc8*Δ, *hob1*Δ and *bzp4*Δ mutants exhibited greatly reduced melanin production; the reduction was similar to that of the *cuf1*Δ mutant. Our results show that Hob1 and Fzc8 promote the expression of *LAC1*, which is the major laccase involved in melanin synthesis^22^, under glucose-starvation conditions, whereas Bzp4 and Cuf1 are not directly involved in *LAC1* expression (Fig. 4c). By contrast, the deletion of *HLH1*, *HLH2*, *YAP1* and *FZC1* greatly enhanced melanin production, although only Hlh1 negatively regulated *LAC1* expression (Fig. 4c).

Another crucial virulence factor is urease, a nickel-dependent protein complex (Ure1, Ure4, Ure6 and Ure7) that converts urea into ammonia, which serves as a nitrogen source. Urease is necessary for sequestration of the pathogen in brain microcapillary beds and crossing the blood–brain barrier through the disruption of tight junctions^23^,^24^. We determined that 19 TFs are involved in either positively or negatively regulating urease production (Supplementary Fig. 6). In summary, our systematic analysis identified a plethora of novel TFs involved in the production of virulence factors in *C. neoformans*.

### TFs modulating antifungal drug and stress responses

For the treatment of cryptococcosis, amphotericin B (AmpB) with or without flucytosine (5-FC) and fluconazole (FCZ) are widely utilized^2^. However, in addition to the toxic side effects of such drugs, the emergence of antifungal drug-resistant *Cryptococcus* strains have caused serious clinical problems^25^. To identify any TFs involved in antifungal drug resistance, we monitored the alteration of antifungal drug susceptibility among the *C. neoformans* TF mutant strains. Numerous TFs were found to be involved in antifungal drug resistance (Supplementary Table 1), implying that *Cryptococcus* can potentially adapt to current antifungal drugs in versatile manners. In response to FCZ, mutants of 55/155 TFs (35.5%) exhibited either increased susceptibility (35 TFs) or resistance (20 TFs), suggesting that azole resistance could readily occur through the modulation of diverse TFs. However, in response to AmpB, mutants of 55 genes exhibited differential susceptibility, with most (47 TFs) showing increased susceptibility and only 8 TF mutants exhibiting increased resistance (Supplementary Table 1). These data support the clinical observation that compared with azole resistance, polyene resistance is rarely observed. Furthermore, supporting the observation that the 5-FC readily elicits the development of drug-resistant strains^26^, our results showed that 27 TFs differentially regulate flucytosine resistance (Supplementary Table 1).

**Figure 5:**
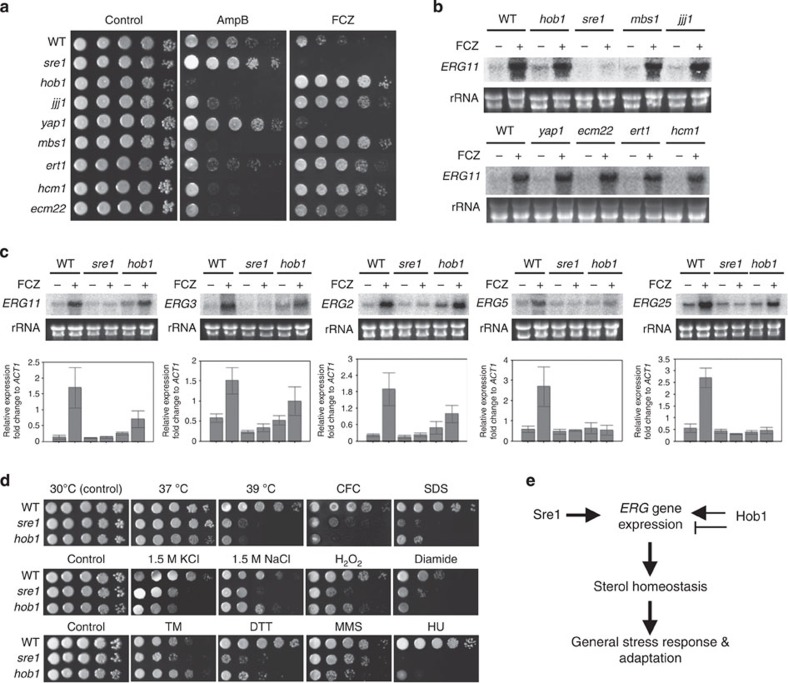
TFs regulating sterol biosynthesis genes govern general environmental stress responses and adaptation in *C. neoformans*. (**a**) TF mutants showing opposite patterns of susceptibility to fluconazole (FCZ) and amphotericin B (AmpB). WT strain H99 and TF mutants grown overnight were 10-fold serially diluted (1 to 10^4^), and then spotted onto YPD containing the following antifungal drugs: 15 μg ml^−1^ FCZ or 1.5 μg ml^−1^ AmpB. (**b**) Northern blot analysis was performed with a *ERG11*-specific probe and the total RNA in cells treated or not treated with 10 μg ml^−1^ FCZ. (**c**) Northern blot assay was performed with *ERG* gene-specific probes. Quantitative reverse transcription–PCR analysis was performed with each *ERG* gene-specific primer using complementary DNA synthesized from the total RNA in cells treated or not treated with 10 μg ml^−1^ FCZ. Duplicate technical experiments with two or more biological samples were performed. Representative images from independent experiments for each *ERG* gene are shown. Error bars indicate s.d. (**d**) The role of Sre1 and Hob1 in environmental stress responses and adaptations. Strains grown as described in **a** were spotted onto YPD containing the stress-inducing agents: 3.5 mM H_2_O_2_, 2.5 mM diamide, 0.3 μg ml^−1^ tunicamycin (TM), 15 mM dithiothreitol (DTT), 5 mg ml^−1^ calcofluor white (CFW), 0.03% SDS, 0.04% methyl methanesulfonate (MMS) or 100 mM hydroxyurea (HU). (**e**) The proposed model for the role of Sre1 and Hob1 in the sterol homeostasis and general stress responses of *C. neoformans.*

We noted that the deletion of some TFs regulated azole and polyene susceptibility in an opposite manner (Fig. 5a; Supplementary Table 1), possibly because these might directly control *ERG11* expression and sterol biosynthesis and affect polyene-binding capacity. Two of these TFs were previously reported to be Erg11 regulators. Sre1, a key sterol regulatory TF, forms a complex with Scp1 as a part of the sterol regulatory element-binding protein pathway in *C. neoformans*^27^,^28^. Mbs1 negatively regulates basal *ERG11* expression and therefore its deletion increases azole resistance but decreases polyene resistance in *C. neoformans*^7^. To further test whether other TFs are also involved in *ERG11* regulation, we measured *ERG11* expression levels in these TF mutants under both sterol-replete and -depleted conditions (Fig. 5b). As expected, basal and induced *ERG11* levels were substantially lower in the *sre1*Δ mutant than in the WT strain. Notably, deleting *HOB1* markedly increased the basal expression levels of *ERG11*. To determine whether Hob1 is involved in the regulation of other *ERG* genes, we monitored the expression of *ERG2*, *ERG3*, *ERG5* and *ERG25* in the WT, *hob1*Δ and *sre1*Δ strains under sterol-replete and -depleted conditions. The expression of all of these *ERG* genes was induced in response to sterol depletion through FCZ treatment in the WT strain, but not in the *sre1*Δ strain (Fig. 5c). Deletion of *HOB1* markedly induced the basal expression of *ERG2* (Fig. 5c). By contrast, under sterol depletion, the induction of *ERG2*, *ERG3*, *ERG5*, *ERG11* and *ERG25* was decreased in the *hob1*Δ mutant (Fig. 5c). The tight regulation of *ERG* expression appeared to be mostly absent in the *hob1*Δ mutant, indicating that Hob1 is a key regulator of ergosterol gene expression.

Notably, the TFs involved in sterol biosynthesis also appeared to be involved in environmental stress responses and adaptation, which are critical for the survival and proliferation of *C. neoformans* within the host because the pathogen encounters drastic environmental changes during infection^3^. Reflecting diverse types of external stresses, 145 TFs were identified to be involved in sensing and responding to at least one type of stress (Fig. 2; Supplementary Fig. 7). Among these TFs, the two sterol regulators, Sre1 and Hob1, appeared to be general stress-responsive TFs that govern multiple stress responses and adaptations. Strikingly, the deletion of *HOB1* or *SRE1* substantially reduced resistance to osmotic/salt, oxidizing/reducing, genotoxic, endoplasmic reticulum (ER) and cell wall/membrane stresses (Fig. 5d). The *hob1*Δ and *sre1*Δ mutants exhibited similar stress resistance/susceptibility patterns under most of the tested environmental stresses, and this is in stark contrast to their opposite resistance patterns towards FCZ and AmpB; this result strongly suggested that sterol homeostasis is critical for controlling stress response and adaptation in *C. neoformans* (Fig. 5e).

### TFs affecting infectivity and virulence of *C. neoformans*

Identification of TFs required for the pathogenicity of *C. neoformans* is critical for future development of novel antifungal drugs and therapeutic methods. Here, we employed two large-scale assays: (1) a virulence assay conducted in the invertebrate insect larval model system *Galleria mellonella*; and (2) a STM-based infectivity assay conducted in a murine inhalation model. These assays using one insect host and one mammalian host model have been widely adopted for large-scale virulence/infectivity assays in other fungi as well as *C. neoformans*^10^.

**Figure 6:**
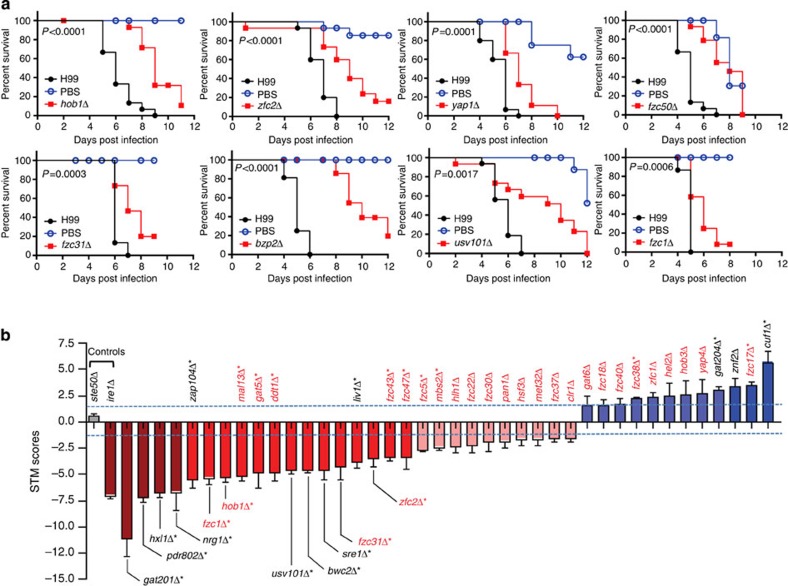
TFs involved in the pathogenicity of *C. neoformans*. (**a**) Novel virulence-regulating TFs identified using the *G. mellonella* killing assay. *P* values shown in the graph were calculated using the Log-rank test to measure statistical differences between the WT strain (H99) and each TF mutant strain. The insect infected with a second independent mutant for each TF exhibited similar survival patterns (Supplementary Fig. 8). (**b**) Virulence-regulating TFs identified using the signature-tagged mutagenesis (STM)-based mouse study. The *y* axis indicates the average STM scores from two independent mutants for each TF. The STM score for each mutant was calculated using quantitative PCR performed using a common primer and the signature-tag-specific primers listed in Supplementary Data 2. The *ste50*Δ and *ire1*Δ mutants were used as virulent and non-virulent control strains, respectively, based on previous reports^9^,^55^. Blue-dotted lines indicate cutoffs (±1.5) that we set to define meaningful changes in virulence based on the STM scores of the *ste50*Δ mutant. Mutants illustrated in red are reported for the first time in this study. Virulence-related TFs were divided into two groups. In the high-confidence group (marked as an asterisk (*)), TF mutants exhibit high average STM scores (>2 or <−2) and each STM score of two independent mutants is statistically significant (*P<*0.05 compared with *ste50*Δ mutant) and beyond the cutoff. In the remaining low confidence group, the average STM scores for TF mutants range from −2.0 to −1.5 or 1.5 to 2.0 with significant *P* values (<0.05), but one of STM scores from two independent mutants could be within the cutoff, although both STM scores have a similar trend (both positive or negative values). The *P* value between control and mutant strains was calculated by one-way analysis of variance with Bonferroni's multiple-comparison test with three biological replicates (three mice per set of experiments). Error bars indicate s.e.m.

Using the insect-based virulence assay, we confirmed that 17 TF genes are involved in the virulence of *C. neoformans* (Fig. 6a; Supplementary Fig. 8; Supplementary Table 2). The mutants identified include nine TF mutants (*hxl1*Δ, *ada2*Δ, *sre1*Δ, *nrg1*Δ, *bwc2*Δ, *crz1*Δ, *pdr802*Δ, *gat201*Δ and *gat204*Δ) that were previously reported to show reduced virulence in a murine model of systemic cryptococcosis^9^,^10^,^18^,^27^,^28^,^29^,^30^,^31^,^32^. This further indicated a strong correlation between the insect and murine models in terms of the pathogenicity of *C. neoformans*. Besides the deletion of these known TFs, deletion of *HOB1, BZP2*, *USV101*, *YAP1*, *ZFC2*, *FZC1*, *FZC50* and *FZC31* significantly reduced the virulence of *C. neoformans*.

**Figure 7:**
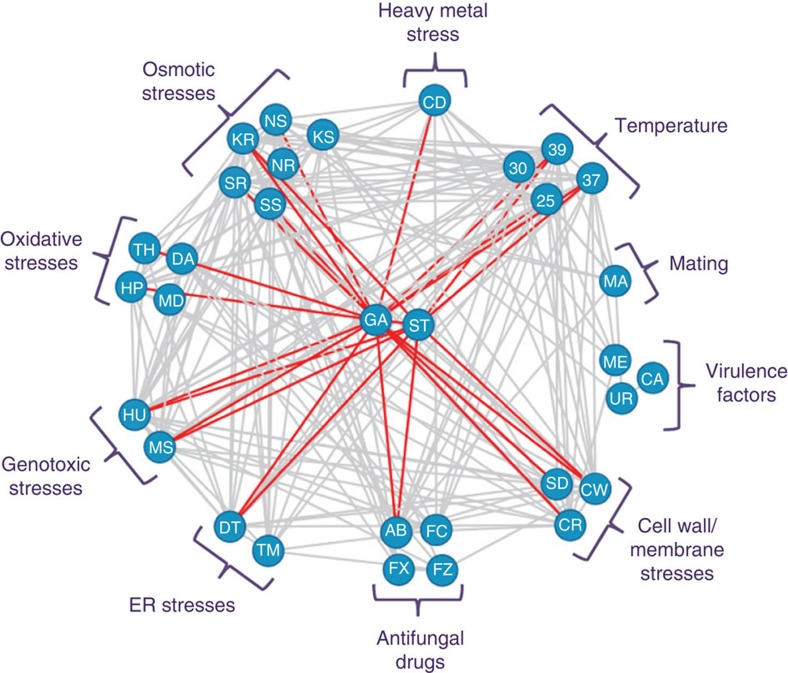
Functional correlation between pathogenicity and phenome data. Functional correlation was calculated using Pearson correlation coefficient (PCC) scores obtained from the results of tested phenotypes. Red lines indicate correlation between virulence and tested phenotypes. Grey lines indicate correlation among in vitro phenotypes. The abbreviations used are the same as those in the phenome heat map in Fig. 2, except for GA (*G. mellonella* virulence data) and ST (signature-tagged mutagenesis data). When the 23 highly confident virulence-related TFs from ST (Fig. 6b) were compared with the 17 virulence-related TFs from GA (Fig. 6a), the PCC score was 0.5359 (*P<*0.0001). When the total of 40 virulence-related TFs from the ST analysis (Fig. 6b) were compared with the 17 virulence-related TFs from GA, the PCC score was 0.4782 (*P<*0.0001).

Using the STM-based murine infectivity assay, we identified 40 virulence genes (Fig. 6b; Supplementary Fig. 9; Supplementary Table 2). The STM score for each mutant was calculated based on the quantitative PCR score=Log_2_(output/input) in the lung from the sacrificed mice (average score from three mice). Among all the sets studied, the *ire1*Δ mutant, which is a non-virulent control strain, exhibited a highly reduced STM score (−7.05±1.49), whereas the *ste50*Δ mutant, a virulent control strain, showed an STM score of 0.52±1.07. Further supporting the quality of the STM assay, 11/40 TFs identified here were previously reported to be involved in virulence^9^,^10^,^29^,^30^. The *gat201*Δ and *pdr802*Δ mutants exhibited drastically reduced STM scores (−11.125 and −7.212, respectively) as reported^10^. Similarly, the STM scores of the *zap104*Δ and *liv1*Δ mutants were also decreased (−5.528 and −3.875, respectively)^10^. Furthermore, the *hxl1*Δ, *nrg1*Δ and *bwc2*Δ mutants also showed highly reduced STM scores. The 11/40 TFs identified by the STM analysis (*GAT201*, *PDR802*, *HXL1*, *BWC2*, *NRG1*, *FZC1*, *HOB1*, *USV101*, *ZFC2*, *SRE1* and *FZC31*) were also discovered using the insect model. The virulence assay data from the insect model were statistically significantly correlated with the STM-based infectivity data from the murine model based on the Pearson correlation coefficient (PCC) analysis (Fig. 7). Among the novel virulence-related TFs that were screened using only the STM-based murine model, the *fzc31*Δ and *ddt1*Δ mutants exhibited highly reduced STM scores (−4.328 and −4.832, respectively). Our phenome database revealed that the *fzc31*Δ mutant exhibited increased susceptibility to osmotic, oxidative and cell membrane stresses, which might collectively affect virulence. By contrast, the only notable phenotype observed in the case of the *ddt1*Δ mutant was a weak dithiothreitol (DTT) sensitivity, which is not likely responsible for the marked decrease in the survival of the mutant in the lung because several other DTT-sensitive TF mutants were as virulent as the WT strain. Therefore, Ddt1 is likely to modulate one or more virulence factors of *C. neoformans* that were not addressed in this study.

To further understand the correlation among phenotypic traits and virulence, we measured the degree of linear dependence by calculating all PCCs between two possible *in vitro* and *in vivo* phenotypic combinations tested in this study; these correlations are illustrated in a combined network (Fig. 7). The correlation network revealed that the virulence of *C. neoformans* is highly correlated to growth at distinct temperatures and osmotic and cell wall/membrane-stress responses and moderately related to oxidative, genotoxic and ER stress responses. Notably, these stress phenotypes were also highly inter-correlated (Fig. 7), suggesting that several core stress signalling networks might exist, including the known Hog1, Pkc1/Mpk1 and UPR pathways. By contrast, mating and resistance to antifungal drugs, except to resistance to AmpB, were not significantly related to the virulence of *C. neoformans*. The production of virulence factors did not appear to be correlated to *in vivo* virulence, and this is likely because increased virulence-factor production often did not result in increased virulence. Supporting this, reduced capsule production was found to be highly correlated to reduced virulence by PCC analysis (*P<*0.05).

## Discussion

In this study, we constructed 322 gene-deletion strains representing 155 TFs to systematically analyse their *in vitro* and *in vivo* phenotypic traits in *C. neoformans*. Although large-scale TF phenome data are available for *S. cerevisiae*, *Schizosaccharomyces pombe*, *C. albicans*, *Candida glabrata*, *Fusarium graminearum* and *Neurospora crassa*^11^,^33^,^34^,^35^,^36^, all of these are ascomycetes that phylogenetically diverged from the basidiomycetes at least 500 million years ago^37^, and only a limited number of *C. neoformans* TF genes appear to have phylogenetically relevant orthologues in the ascomycete model fungi. As we excluded general TFs and sequence-nonspecific DNA-binding proteins, this is likely not a complete set of TFs but represents a majority of the sequence-specific DNA-binding TFs present in the *C. neoformans* genome, and as such the data presented here provide comprehensive insights into transcriptional networks in diverse basidiomycetous fungi and human fungal pathogens.

Our *Cryptococcus* TF mutant library and its phenome database have an exceptionally high functional coverage (93%) compared with those performed in other pathogenic ascomycetous fungal species. Homann *et al.*^11^ constructed 166 TF mutants in *C. albicans* and tested their phenotypic traits under 50 different growth conditions and found that over the 50% of them exhibit at least one moderate phenotype. Son *et al.*^36^ constructed 657 TF mutants in *F. graminearum* and analysed their phenotypic traits under 17 different growth conditions. Interestingly, however, only 26% of them displayed discernible phenotypes^36^. Schwarzmüller *et al.*^35^ constructed 619 deletion mutants containing 177 TF deletion mutants in *C. glabrata* and found that about 32% of them exhibited at least one discernible phenotypic trait. Therefore, our high functional coverage TF phenome analysis not only led to the discovery of novel phenotypic traits of previously characterized TFs, but also identified several novel TFs involved in diverse biological aspects of *C. neoformans*.

Out of *C. neoformans*’s 178 TFs, only a limited number of orthologues are present in the model yeasts and fungi: 53 in *S. pombe*, 68 in *N. crassa*, 82 in *F. graminearum*, 64 in *C. albicans*, 51 in *S. cerevisiae* and 53 genes in *C. glabrata* (Supplementary Data 5). Among them, 22 TFs (Esa1, Top3, Cef1, Rsc8, Pzf1, Mig1, Rum1, Jjj1, Ada2, Mbs1, Fab1, Hel2, Gat1, Miz1, Skn7, Mcm1, Liv4, Crz1, Usv101, Cuf1, Mbf1 and Rlm1) are conserved and considered to be core fungal TFs. Supporting their core functions, six of them (Esa1, Top3, Cef1, Rsc8, Pzf1 and Mig1) are essential in *S. cerevisiae* and four of them (Esa1, Cef1, Rsc8 and Pzf1) also appeared to be essential in *C. neoformans*. In this study, we constructed mutants for 16 out of the 22 core TFs and discovered that their phenotypes cover a broad range of biological processes. Strikingly, the finding that nine of them (Jjj1, Ada2, Mbs1, Gat1, Skn7, Mcm1, Usv101, Crz1 and Cuf1) are involved in virulence-factor production (capsule, melanin and urease) and virulence suggests that *C. neoformans* evolved several core TF networks into pathogenicity-related signalling networks.

Common phenotypic traits examined by this and other studies in the systematic analyses of fungal TF networks include development and differentiation. For 103 TFs studied in *N. crassa*, 88 of them have orthologues in the *F. graminearum* genome^33^,^36^. Among them, only nine mutants exhibit similar phenotypes in differentiation processes of both fungi. By contrast, only 13 out of 103 *N. crassa* TFs have recognizable orthologues in the *C. neoformans* genome. Interestingly, however, nine of them are involved in differentiation of *Neurospora* and six of them (*STE12*, *ATF1*, *BWC2*, *GAT201*, *HOB7* and *SRE1*) were shown to play a role in sexual differentiation in *C. neoformans.* Furthermore, 27 of 82 *F. graminearum* TFs, which are highly homologous to *Cryptococcus* TFs, are involved in sexual development of the plant fungal pathogen and 10 of them (Hob7, Hcm1, Znf2, Gat1, Fzc26, Zap104, Ada2, Rum1, Mcm1 and Usv101) also exhibited altered mating response in *C. neoformans*. Therefore, among many phenotypic traits, some genes involved in sexual differentiation appeared to be functionally well conserved among fungi, although each fungus also contains a unique set of TFs to govern its own differentiation processes.

In fact, the first large-scale functional analysis of *C. neoformans* genes has been previously performed by Liu *et al.*^10^, who constructed deletion mutants for 1,201 genes, including 58 TFs, and analysed three virulence-related *in vitro* phenotypic traits (capsule, melanin and growth at 37 °C) and *in vivo* pulmonary infectivity. However, their mutant collection consists of only one PCR-confirmed mutant strain for each gene in the background of the CMO18 strain, which is virulence-attenuated, nearly sterile H99 strain^4^,^10^. By contrast, our TF mutant collection consists of two independent Southern blot analysis-confirmed mutants for all 155 TFs in the background of the H99S strain that retains full virulence and fertility^6^. For parallel phenotypic comparison, we reconstructed 53 out of their CMO18 58 TF mutants in the H99S strain background. Due to differences in strain background and experimental conditions, we found only partial overlap between the two *in vitro* phenotypic analyses. Melanin-regulating TFs identified by this study were not discovered by Liu *et al.* because the CMO18 strain is defective in melanin production and they used different melanin-inducing media. As the two studies also used two different capsule-inducing media, only one TF, Gat201, was commonly found to be highly defective in capsule production by these two studies. Our study identified numerous novel TFs orchestrating production of capsule and melanin. Particularly, we found that Hob1, Fzc8 and Hlh1 are involved in regulation of *LAC1* expression. By contrast, we found a significant overlap in the STM-based murine infectivity data between two studies; out of 17 infectivity-related TF reported by Liu *et al.*, 8 of them (Zap104, Znf2, Nrg1, Liv1, Gat201, Bwc2, Fzc38 and Pdr802) were similarly identified by this study, suggesting a strong correlation in terms of the infectivity phenotype between the two studies.

In this study, we identified novel regulators of sterol biosynthesis in *C. neoformans* in addition to a known sterol regulator, Sre1. In particular, the homeobox protein Hob1 appears to function as a key regulator of sterol biosynthesis by affecting multiple ergosterol biosynthesis genes. Our data suggest that Hob1 must effectively repress sterol biosynthesis genes under sterol-replete conditions. Without this control, cells might not be able to adapt to environmental changes, as indicated by our finding that the *hob1*Δ mutants exhibited patterns of extreme stress sensitivity to multiple stresses that were similar to the sensitivity patterns exhibited by the *sre1*Δ mutant. In *S. cerevisiae*, Mot3 (a C2-H2 zinc-finger TF) and Rox1 (an HMG domain TF), which are not homologous to Hob1, play similar repressive roles in sterol biosynthesis in a HOG pathway-dependent manner^38^, suggesting that sterol biosynthesis in *C. neoformans* appears to be governed by a mechanism that is considerably distinct from that in *S. cerevisiae*.

Another key finding of this study with clinical and pharmaceutical implications is the discovery of an unprecedented number of TFs involved in virulence of *C. neoformans*, including several structurally and functionally unique TFs. Although TFs are considered to be less optimal drug targets in general, it is not impossible to develop TF-specific drugs as witnessed in the development of a STAT4 inhibitor, Lisofylline, for treating diabetes and STAT3 inhibitors for cancer treatment^39^,^40^ and in the small-molecule-mediated inhibition of FOXM1 transcriptional programme^41^. Here we determined that 45 genes (32 novel and 13 known TFs) are involved in pathogenicity of *C. neoformans* based on the insect-based virulence assay and STM-based murine infectivity assay, which are two complementary assays for large-scale screening of potential virulence factors^10^,^42^. Particularly, the virulence-related TFs confirmed using both approaches warrant particular attention. Besides recognized TFs, these include novel TFs, such as Usv101, Hob1, Fzc1, Fzc31 and Zfc2. Virulence defects observed in the *hob1*Δ and *usv101*Δ mutants might result from increased thermosensitivity and severe defects in melanin production and multiple stress responses. By contrast, obvious virulence-related phenotypic traits were not detected in the *zfc2*Δ, *fzc1*Δ and *fzc31*Δ mutants. The *fzc1*Δ mutant in particular exhibited highly enhanced capsule and melanin production. Therefore, these TFs might function in the production of other virulence factor(s), which could include capsule-independent antiphagocytic factor^32^, phospholipases^43^, inositol uptake and metabolic systems^44^ and giant/titan cell formation^45^. Therefore, an expanded phenotypic profiling of the TF mutant library will further reveal the molecular functions of each virulence-related TF in the pathogenicity of *C. neoformans*.

When virulence assay data in human pathogenic fungi from this and others studies were compared, we found several evolutionarily conserved virulence-regulating TFs, which could be exploited as broad-spectrum antifungal drug targets. When two large-scale virulence analyses of *C. neoformans* and *C. albicans* TF mutants^11^ were compared, the five TFs (Nrg1, Rim101, Crz1, Usv101 and Zap104) appear to be commonly involved in virulence of the two fungal pathogens. Crz1 is also known to be required for the virulence of *A. fumigatus* and *C. glabrata*^46^,^47^. Comparison of our virulence–phenotype correlation network showed that *in vivo* virulence of *C. neoformans* is highly correlated to stress responses, but not to mating and differentiation. By contrast, morphological switching and differentiation processes appear to be highly correlated with virulence of other fungal pathogens. In *F. graminearum*, sexual development is highly correlated to virulence^36^. In *C. albicans*, Noble *et al.*^48^ identified 115 infectivity-attenuated mutants, about 40% of which exhibits altered morphological switching and proliferation. Although sexual differentiation was not connected to virulence of *C. neoformans*, other types of morphological changes could be related to virulence. The production of titan cells, which is the only known morphological switching event occurring during host infection, is required for full virulence of *C. neoformans*^49^,^50^. As our study has not monitored the role of each TF in titan cell formation systematically, it is possible that TF networks governing morphogenesis could be critical for virulence. Recently, two TFs, Rim101 and Mbs1 were found to be involved in titan cell formation^7^,^51^. Future screening of titan cell-regulating TFs could significantly correlate morphogenesis to virulence in *C. neoformans*.

In conclusion, the current systematic functional profiling of transcriptional networks will enhance our ability to comprehensively understand the complex signalling networks that govern the general biology and pathogenicity of *C. neoformans*.

## Methods

### Ethics statement

Animal care and all experiments were conducted in accordance with the ethical guidelines of the Institutional Animal Care and Use Committee (IACUC) of Yonsei University. The Yonsei University IACUC approved all of the vertebrate studies.

### Construction of the *C. neoformans* TF mutant library

TF mutant strains were constructed in the *C. neoformans* serotype A H99S strain background. Gene-disruption cassettes containing the nourseothricin-resistance marker (*NAT*) and signature-tagged sequences were generated using overlap PCR or *NAT-*split marker/double-joint PCR strategies^52^,^53^. All of the primers used in this study are listed in Supplementary Data 2. In the overlap PCR method, the 5′- and 3′-flanking regions of the TF genes were amplified by using primers L1 and L2 and primers R1 and R2, respectively, together with H99 genomic DNA in the first round of PCR. Primers M13Fe (M13 forward extended) and M13Re (M13 reverse extended) were used for amplifying the dominant selectable marker *(NAT)* containing unique signature-tagged sequences. In the second round of PCR, the TF gene-disruption cassettes were generated by means of overlap PCR performed using primers L1 and R2 and the first-round PCR products as templates. In the double-joint PCR method, the 5′- and 3′-flanking regions of the TF genes were amplified using, respectively, the primer pairs L1/L2 and R1/R2 with H99 genomic DNA in the first round of PCR. The 5′- and 3′-regions of *NAT*-split markers were amplified using primers M13Fe and NSL and primers M13Re and NSR, respectively, together with pNATSTM, which harboured unique signature-tagged sequences. The amplified gene-disruption cassettes were combined with 600 μg of gold microcarrier beads (0.6 μm, Bio-Rad) and were introduced into the H99S strains using the biolistic transformation apparatus^54^. Stable nourseothricin-resistant transformants were initially screened by means of diagnostic PCR. The accuracy of the genotypes of the positive transformants was validated by means of Southern blot analysis. *Cryptococcus* genomic DNA was extracted using the CTAB (cetyl trimethyl ammonium bromide) method^55^. Isolated genomic DNA from each TF mutant was digested with the indicated restriction enzyme. The digested genomic DNAs were separated by 1% agarose gel electrophoresis. The agarose gel was transferred into the denatured buffer containing 0.5 M NaOH and 1.5 M NaCl for 45 min. Next, the agarose gel was transferred into the neutralization buffer containing 1.5 M NaCl and 0.5 M Tris adjusted with pH 8 for 45 min. The digested genomic DNA were transferred to the nylon membrane using 10 × SSC buffer and fixed by 1,200 J m^−2^ ultraviolet exposure. The membrane was hybridized with a gene-specific and radioactively labelled probe using modified church hybridization buffer (1 mM EDTA, 0.25 M Na_2_HPO_4_, 1% hydrolysated casein, 7% SDS, 6% H_3_PO_4_) overnight. The membrane was washed for 15 min with washing buffer 1 (2 × SSC and 0.1% SDS) and washing buffer 2 (1 × SSC and 0.1% SDS). Next, the membrane was exposed to autography film for 1 day. All TF mutant strains were deposited in the Korean Culture Collection of Microorganisms in Korea and Center of Microbial Pathogenesis at Duke University in USA.

### Growth and chemical susceptibility analyses

For analysing growth phenotypes at distinct temperature, we monitored the growth of each mutant at a range of temperatures (25, 30, 37 and 39 °C) on agar-based yeast extract-peptone dextrose (YPD) medium. For analysing stress-related phenotypes and antifungal drug susceptibility, cells grown at 30 °C in liquid YPD medium for 16 h were 10-fold serially diluted (1 to 10^4^ dilutions) and spotted on YPD medium containing the indicated concentrations of the following chemicals: osmotic (sorbitol) and cation/salt stresses (NaCl and KCl) under either glucose-rich (YPD) or glucose-starved (yeast extract-peptone, YP) conditions; oxidative stress (hydrogen peroxide (H_2_O_2_), *tert*-butyl hydroperoxide (an organic peroxide), menadione (a superoxide anion generator), diamide (a thiol-specific oxidant)); heavy-metal stress (CdSO_4_); genotoxic stress (methyl methanesulfonate and hydroxyurea); cell membrane/wall-destabilizing stress (SDS, calcofluor white and Congo red); ER stress (tunicamycin and DTT); and antifungal agents (fludioxonil, FCZ, AmpB and flucytosine). Cells were incubated at 30 °C and photographed for 2–5 days.

### Mating, cell fusion and pheromone gene expression assay

For analysing mating phenotypes, we set up unilateral mating crosses by coculturing each TF mutant (the serotype A *MAT*α strain) with serotype A *MAT***a** WT KN99**a** strain. Each strain was cultured in YPD medium at 30 °C for 16 h and equal concentration of cells (10^7^ cells per ml) were mixed, spotted onto V8 mating media (pH 5) and incubated in a dark at room temperature for 1–2 weeks. Filamentous growth was monitored weekly and photographed using an Olympus BX51 microscope equipped with a SPOT Insight digital camera (Diagnostic Instrument Inc.). For the cell fusion assay, each *MAT*α TF mutant or control strain (YSB119) containing *NAT* and *MAT***a** control strain (YSB121) containing neomycin-resistant marker were cultured at 30 °C in liquid YPD medium for 16 h and the concentration of cells was adjusted to 10^7^ cells per ml with distilled water. Each *MAT*α and *MAT***a** strain were mixed in an equal volume, spotted onto V8 medium and incubated in a dark at room temperature for 24 h. Then the cells were scraped, resuspended in 1 ml distilled water and spread onto YPD medium containing both nourseothricin (100 μg ml^−1^) and G418 (50 μg ml^−1^). The plates were further incubated at 30 °C and the number of colonies on each plate was determined. For monitoring pheromone gene expression, the *MAT*α and KN99**a** strains were mixed with equal concentration of cells (10^8^ cells per ml), spread onto the V8 medium and incubated in the dark at room temperature for 18 or 24 h. Then cells were scraped, pelleted, frozen in liquid nitrogen and lyophilized overnight for total RNA isolation, followed by northern blot analysis with a specific mating pheromone gene (*MFα1*)-specific probe.

### Expression analysis by northern blot and quantitative RT–PCR

Total RNA was isolated from each sample using Trizol reagent^55^. For northern blot analysis, 10 μg of RNA was separated in 1% agarose gel made with diethyl pyrocarbonate-treated water and 1 × MOPS running buffer by electrophoresis. The gel was washed three times with distilled water, transferred to a nylon membrane using 20 × SSC buffer and fixed by 1,200 J m^−2^ ultraviolet exposure. The membrane was hybridized with a gene-specific and radioactively labelled probe using modified church hybridization buffer. The membrane was washed with the washing buffer 1 and 2, and then the membrane was exposed to autoradiography film for 1–2 days. To analyse the expression of *ERG2*, *ERG3*, *ERG5*, *ERG11* and *ERG25*, we grew WT, *sre1*Δ and *hob1*Δ mutants in liquid YPD medium overnight. The overnight culture was then inoculated in 100 ml of fresh YPD medium at 30 °C and grown until the OD_600_ of the culture reached ∼1.0. To prepare the zero-time sample, 50 ml of cell culture was sampled and the remaining culture was treated with FCZ (final concentration: 10 μg ml^−1^) for 90 min. Total RNA was isolated using TRIzol reagent and complementary DNA was synthesized using M-MuLV reverse transcriptase (Thermo scientific). Northern blot analysis was performed with each *ERG* gene-specific probe that was amplified with *ERG* gene-specific primers with the total RNA in cells treated or not treated with FCZ. Primers: B5789 and B5790 for *ERG2*; B1720 and B1721 for *ERG3*; B671 and B674 for *ERG5*; B678 and B1598 for *ERG11*; B1718 and B1719 for *ERG25*. Quantitative real-time PCR was performed with each gene-specific primer using a MyiQ2 Real-Time PCR detection system (Bio-Rad). Primers: B5789 and B6838 for *ERG2*; B1720 and B6839 for *ERG3*; B671 and B672 for *ERG5*; B677 and B678 for *ERG11*; B2695 and B6840 for *ERG25*; and B679 and B680 for *ACT1*.

### *In vitro* virulence-factor production assay

Capsule production was measured in both qualitative and quantitative manners. Cells were grown at 30 °C in liquid YPD medium for 16 h, spotted onto Dulbecco's Modified Eagle's (DME) solid medium and incubated at 37 °C for 2 days. Then cells were scraped and washed with PBS. For qualitative measurement, capsules were stained by India ink (Bactidrop; Remel) and visualized using an Olympus BX51 microscope equipped with a Spot insight digital camera (Diagnostic Instrument Inc.). For quantitative measurement, the cells collected from DME solid medium were fixed with 10% formalin and an equal number of cells (2.5 × 10^7^ cells per ml) was loaded into a haematocrit capillary tube, which was subsequently placed vertically to allow cells to be packed by gravity for 10 days. The packed cell volume ratio was measured by calculating the ratio of the length of the packed cell volume phase to the length of the total volume phase (cells+medium). The relative packed cell volume of each mutant was measured by calculating the ratio of the mutant packed cell volume ratio to the WT packed cell volume ratio. Triplicate technical experiments with two or more independent strains were performed. Statistical difference in relative packed cell volume was determined by one-way analysis of variance with Bonferroni’s multiple-comparison test using Prism 6 (Graphpad software). For monitoring melanin production, each strain was cultured at 30 °C in liquid YPD medium for 16 h, spotted on Niger seed agar medium containing 0.1 or 0.3% glucose, incubated at 37 °C and photographed daily. The induction of *LAC1* was monitored by northern blot analysis as previously described. For monitoring urease production, each strain was cultured at 30 °C in liquid YPD medium for 16 h, washed with distilled water and an equal number of cell (5 × 10^4^) was spotted onto Christensen’s agar media. Then the plates were incubated for 7–10 days at 30 °C and photographed.

### The insect-based *in vivo* virulence assay

For the insect-based virulence assay, 15 *Galleria mellonella* caterpillars (body weight: 250±50 mg) in the final instar larval stage, reached within 7 days from the day of shipment (Vanderhorst Inc., St Marys, OH, USA), were randomly sorted into each group. Each *C. neoformans* strain was grown overnight at 30 °C in YPD medium, washed three times and resuspended with PBS. We inoculated 4,000 *C. neoformans* cells per larva through the second to last prolegs of larvae using a 100-μl Hamilton syringe equipped with a 10-μl-size needle and a repeating dispenser (PB600-1, Hamilton). As a non-infection control, PBS was injected. After injection, larvae were incubated in Petri dishes in humidified plastic containers and monitored daily. Larvae were considered dead when they displayed no movement when touched. Larvae that transformed into pupae during experiments were censored for statistical analysis. Survival curves were prepared using Prism 6 (GraphPad) and statistically analysed using the Log-rank (Mantel–Cox) test. We first monitored the survival curve for a single mutant strain for each TF gene (total 155) and statistically compared it with that of the WT strain. In the case of TF mutant that showed statistically significant reduction or enhancement of virulence (*P<*0.05; Log-rank test), we examined a second independent strain.

### The STM-based murine infectivity assay

In the STM-based mouse infectivity test, TF strains tagged with 45 distinct signature tags (Supplementary Data 2) were grown at 30 °C in YPD medium, washed three times with PBS and then pooled; the same number of cells of each strain were used after counting cells using a haemocytometer. The *ste50*Δ and *ire1*Δ mutants tagged with STM#282 or STM#169 sequence were used as virulent and non-virulent control strains, respectively, as described previously^9^,^55^. To obtain the input TF genomic DNA library, the pooled TF strains were 10-fold serially diluted, plated on YPD media, incubated at 30 °C for 3 days and collected by scraping for use in isolating genomic DNA. The output TF genomic DNA library was obtained as follows. Seven-week-old female A/Jcr mice (Jackson Laboratory) anaesthetized with intraperitoneal injection of Avertin (2,2,2-tribromoethanol) were infected through intranasal inhalation of 5 × 10^5^ cells (in 50 μl) of the pooled TF mutants and sacrificed with an overdose of Avertin at 15 days post infection. For each set of assays, we used three mice. Two independent mutants for each TF were tested in a separate STM set assay. Lungs were dissected and homogenized in 4 ml of sterile PBS. Each lung-tissue lysate was spread on YPD media containing 100 μg ml^−1^ of chloramphenicol, incubated at 30 °C for 3 days and collected by scraping to isolate output genomic DNA. Both input and output genomic DNAs were extracted using the CTAB method^55^. Quantitative PCR analysis was performed with the various tag-specific primers listed in Supplementary Data 2 using a MyiQ2 Real-Time PCR detection system (Bio-Rad). We used the 2^−ΔΔCt^ method to determine the STM score^10^,^56^. To determine the input of genomic DNA with a specific tag, a ΔCtin (ΔCttag-input) was calculated by comparing the Ct*ACT1* with average of Cttag from genomic DNA of pooled TF strains (Cttag−Ct*ACT1*). To determine the output genomic DNA with a specific tag, a ΔCtout (ΔCttag-output) was calculated by comparing the Ct*ACT1* with average of Cttag from genomic DNA from each lung of the sacrificed mouse (Cttag−Ct*ACT1*). The STM score of each TF mutant was determined as the Log_2_ 2^−ΔΔCt (ΔCtout−ΔCtin)^.

### *Cryptococcus* transcription factor database

The genome and transcriptome data collected for 178 TFs were processed using the protocol of the standardized genome data warehouse in Comparative Fungal Genomics Platform (CFGP 2.0; http://cfgp.snu.ac.kr/)^57^. The TF family name for each gene was described based on the prediction of Fungal Transcription Factor Database (http://ftfd.snu.ac.kr/)^58^. For detailed information of the predicted genes, pre-computed results of eight bioinformatics programs were provided (InterPro scan, Signalp 3.0, PSortII, TargetP, ChloroP, SecretomeP, predictsNLS and TMHMM2)^59^,^60^,^61^,^62^,^63^,^64^,^65^,^66^. To browse genomics contexts together with key biological features, Seoul National University Genome Browser (SNUGB; http://genomebrowser.snu.ac.kr/)^67^ was incorporated for use with the *Cryptococcus* TF Database (http://tf.cryptococcus.org). In the pages of Browse Scaffolds, Browse Gene Models and 3 gene-family browsers, direct links to the SNUGB module were provided. MySQL 5.0.81 (source code distribution) and PHP 5.2.6 were used for administrating the database and developing web interfaces, respectively. Web pages were provided through Apache 2.2.9 web server.

### Construction of Pearson’s correlation networks

We calculated PCC scores by using Prism 5.0 (GraphPad Software Inc.) based on the results of phenotypic tests (strongly resistant phenotype: 3; moderately resistant phenotype: 2; weakly resistant phenotype: 1; WT-like phenotype: 0; weakly sensitive phenotype: −1; moderately sensitive phenotype: −2; and strongly sensitive phenotype: −3). Networks were visualized using Cytoscape software 3.2.0 based on the PCC scores.

### Statistical analyses

Statistical analyses were performed using Prism 6.0 (GraphPad Software Inc.). Statistical difference was determined using Bonferroni’s multiple-comparison test.

## Additional information

**How to cite this article:** Jung, K.-W. *et al.* Systematic functional profiling of transcription factor networks in *Cryptococcus neoformans*. *Nat. Commun.* 6:6757 doi: 10.1038/ncomms7757 (2015).

## Supplementary Material

Supplementary Figures and Supplementary Tables Supplementary Figures 1-9 and Supplementary Tables 1-2

Supplementary Data 1 List of *Cryptococcus neoformans* transcription factors predicted based on the DNA-binding domain database

Supplementary Data 2 List of primers used for constructing the TF mutant library

Supplementary Data 3 BLAST matrix analysis of *C. neoformans* transcription factors

Supplementary Data 4 Complete phenome heat map of *C. neoformans* transcription factors

Supplementary Data 5 Ortholog mapping and comparison of fungal transcription factors by reciprocal BLAST analysis in model yeasts and filamentous fungi
