# Supplementary material for: Systematic functional profiling of transcription factor networks in Cryptococcus neoformans
Source: Nat Commun. 2015 Apr 7;6:6757. doi: 10.1038/ncomms7757 (PMC4391232; doi:10.1038/ncomms7757)
Supplement: Supplementary Figures and Supplementary Tables — Supplementary Figures 1-9 and Supplementary Tables 1-2 [file ncomms7757-s1.pdf]

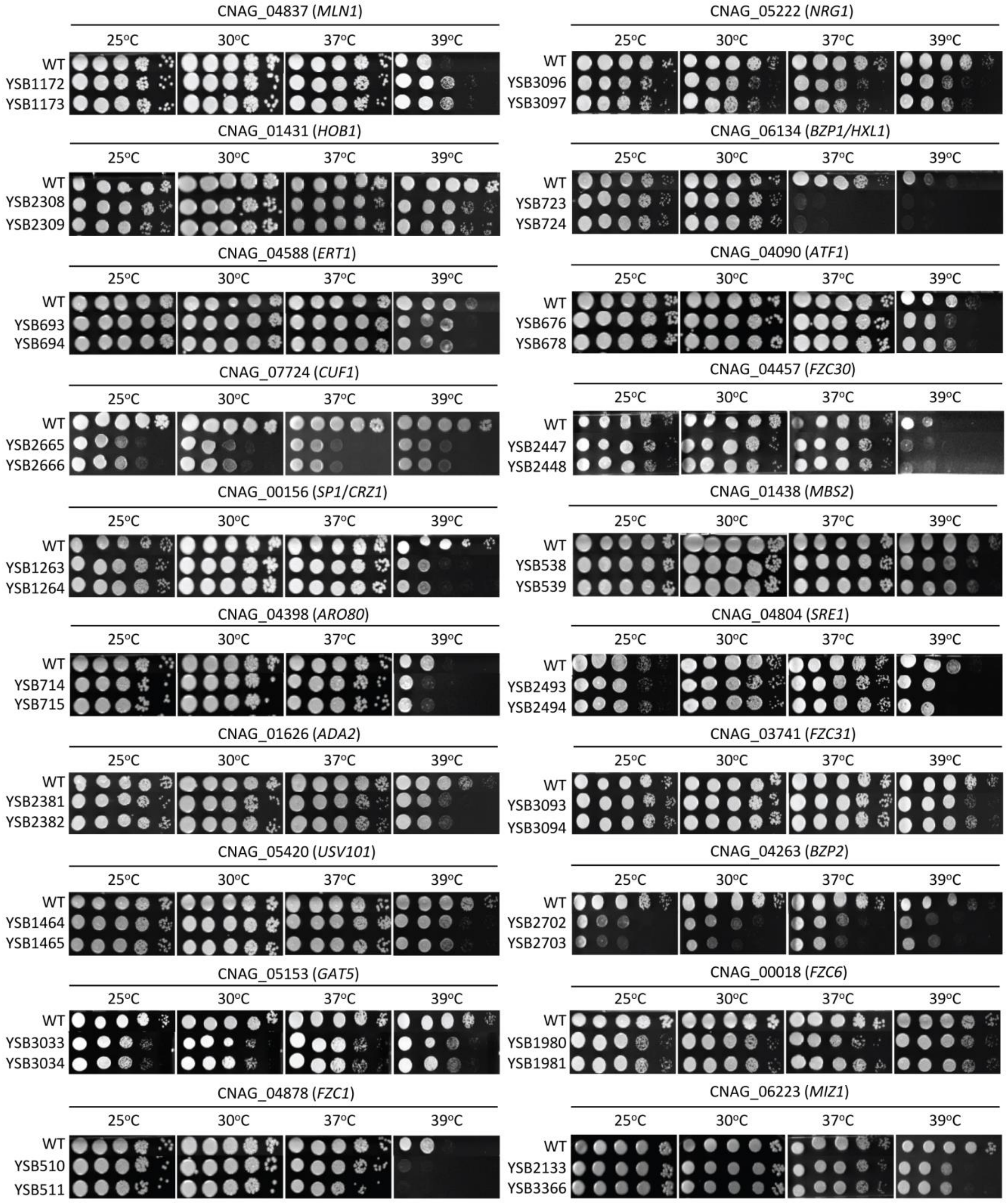
CNAG_04837 (MLN1)
25°C
30°C
37°C
39°C
WT
YSB1172
YSB1173
CNAG_01431 (HOB1)
WT
YSB2308
YSB2309
CNAG_04588 (ERT1)
WT
YSB693
YSB694
CNAG_07724 (CUF1)
WT
YSB2665
YSB2666
CNAG_00156 (SP1/CRZ1)
WT
YSB1263
YSB1264
CNAG_04398 (ARO80)
WT
YSB714
YSB715
CNAG_01626 (ADA2)
WT
YSB2381
YSB2382
CNAG_05420 (USV101)
WT
YSB1464
YSB1465
CNAG_05153 (GAT5)
WT
YSB3033
YSB3034
CNAG_04878 (FZC1)
WT
YSB510
YSB511
CNAG_05222 (NRG1)
WT
YSB3096
YSB3097
CNAG_06134 (BZP1/HXL1)
WT
YSB723
YSB724
CNAG_04090 (ATF1)
WT
YSB676
YSB678
CNAG_04457 (FZC30)
WT
YSB2447
YSB2448
CNAG_01438 (MBS2)
WT
YSB538
YSB539
CNAG_04804 (SRE1)
WT
YSB2493
YSB2494
CNAG_03741 (FZC31)
WT
YSB3093
YSB3094
CNAG_04263 (BZP2)
WT
YSB2702
YSB2703
CNAG_00018 (FZC6)
WT
YSB1980
YSB1981
CNAG_06223 (MIZ1)
WT
YSB2133
YSB3366

Continued

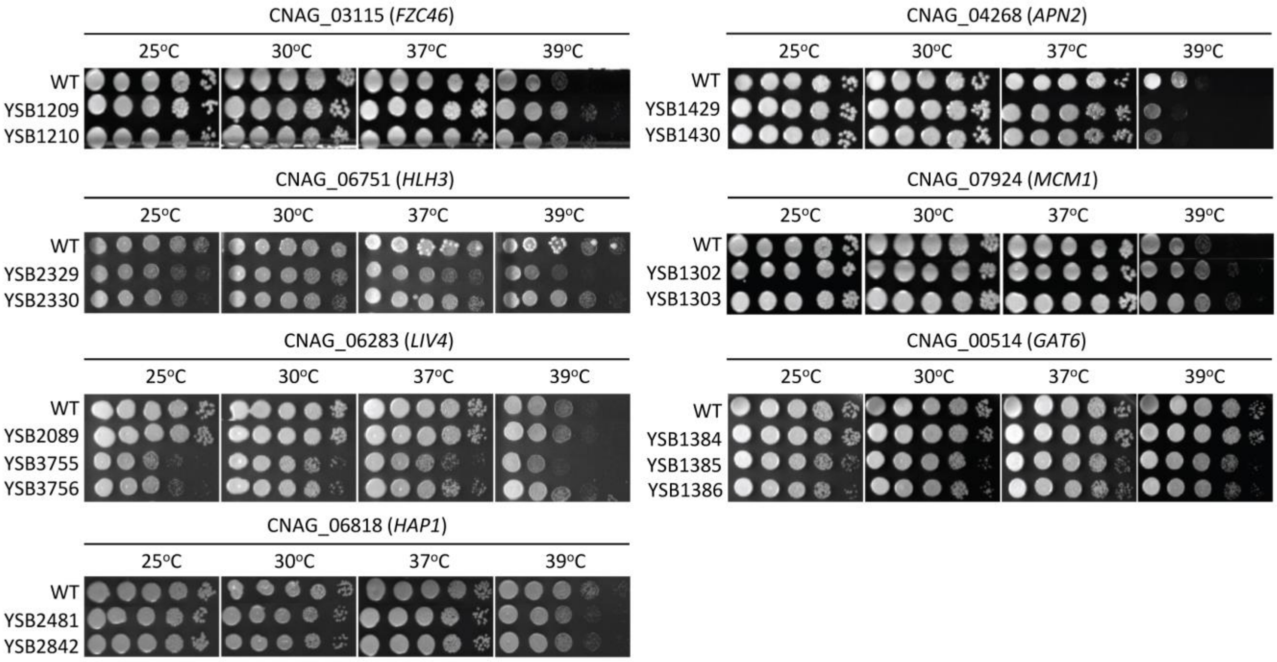

Supplementary Figure 1: Transcription factors required for temperature-dependent growth of *C. neoformans*. *C. neoformans* strains were grown overnight in liquid YPD medium at 30°C, serially diluted (1 to 10^4 dilutions), and spotted (3 µl of each dilution) onto YPD agar medium. Cells were incubated at 25, 30, 37, and 39°C for 3 days and then photographed.

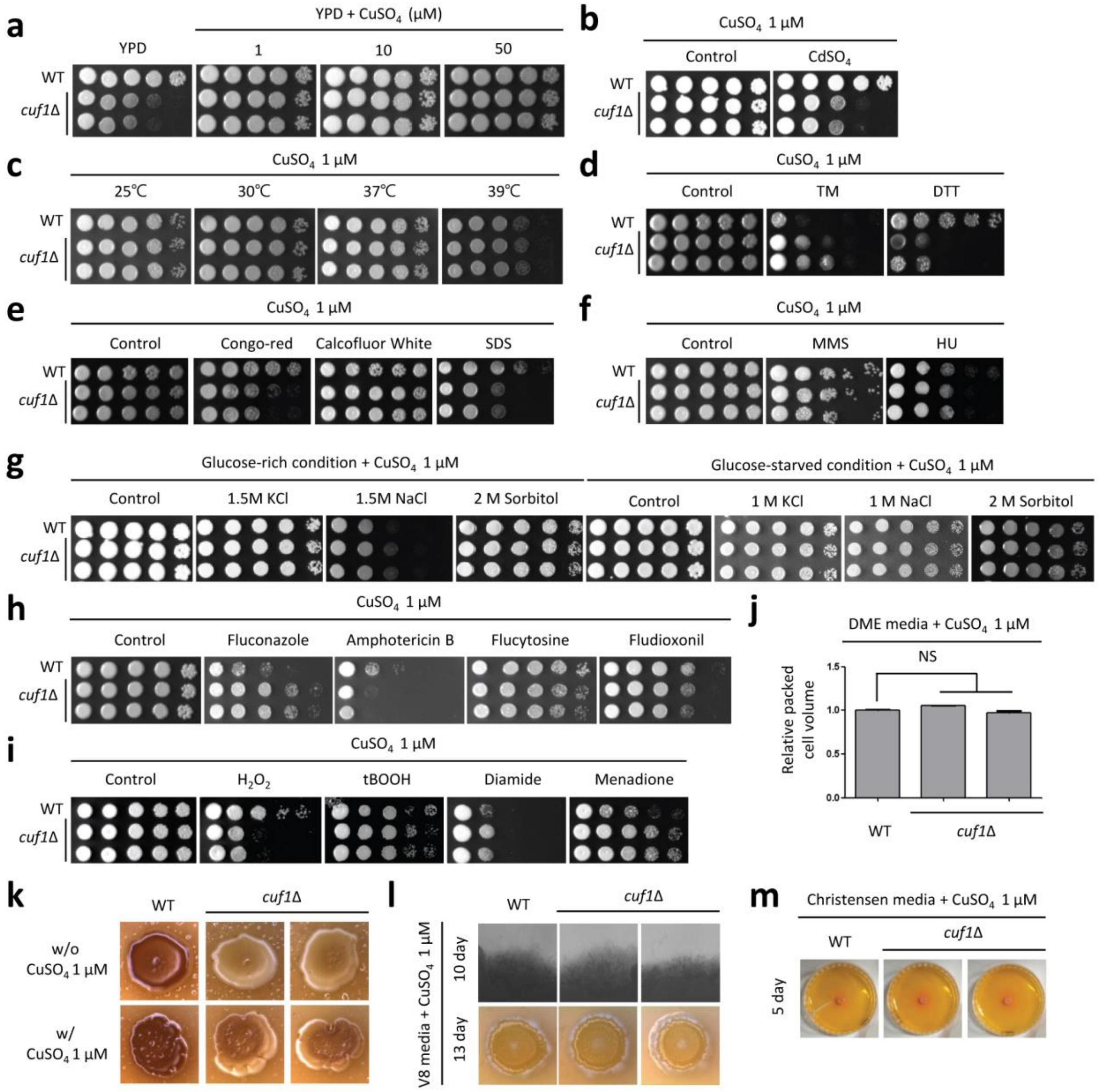

Supplementary Figure 2: The role of Cuf1 and copper uptake in the growth, differentiation, stress responses, antifungal drug susceptibility, and virulence-factor production of *C. neoformans*. (a) WT (H99) and *cuf1*Δ (YSB2665 and YSB2666) mutants were spotted onto YPD media containing the indicated concentrations of $CuSO_4$. Cells were grown at 30°C and photographed after growth for 2 days. (b, d–i) WT and *cuf1*Δ mutants were grown in liquid YPD media for 16 h at 30°C, 10-fold serially diluted (1 to 10^4 dilutions), and spotted (3 µl of each dilution) onto YPD (glucose-rich condition) or YP (glucose-starved condition) agar medium containing 1 µM $CuSO_4$ and the following stress inducers: $CdSO_4$ 30 µM (b), TM 0.3 µg ml^{-1} (c), DTT 15 mM (d), Congo red 0.8% (e), calcofluor white 3 mg ml^{-1} (e), SDS 0.03% (e), MMS 0.03% (f), HU 100 mM (f), NaCl/KCl 1.5 M or sorbitol 2 M (g), fluconazole 14 µg ml^{-1} (h), amphotericin B 0.8 µg ml^{-1} (h), flucytosine 300 µg ml^{-1} (h), fludioxonil 1 µg ml^{-1} (h), H_2O_2 3.5 mM (i), tBOOH 0.8 mM (i), diamide 2.5 mM (i), and menadione 0.02 mM (i). Cells were further incubated at 30°C and photographed daily for 2–4 days. (c) WT and *cuf1*Δ mutants were spotted onto YPD media containing 1 µM $CuSO_4$ and incubated at the indicated temperatures for 2 days. (j–m) To perform the capsule, melanin, mating, and urease assays, cells were spotted or incubated in agar-based Dulbecco's Modified Eagle's (DME) medium, Niger seed medium, V8 medium, and Christensen's agar medium, respectively, containing 1 µM $CuSO_4$

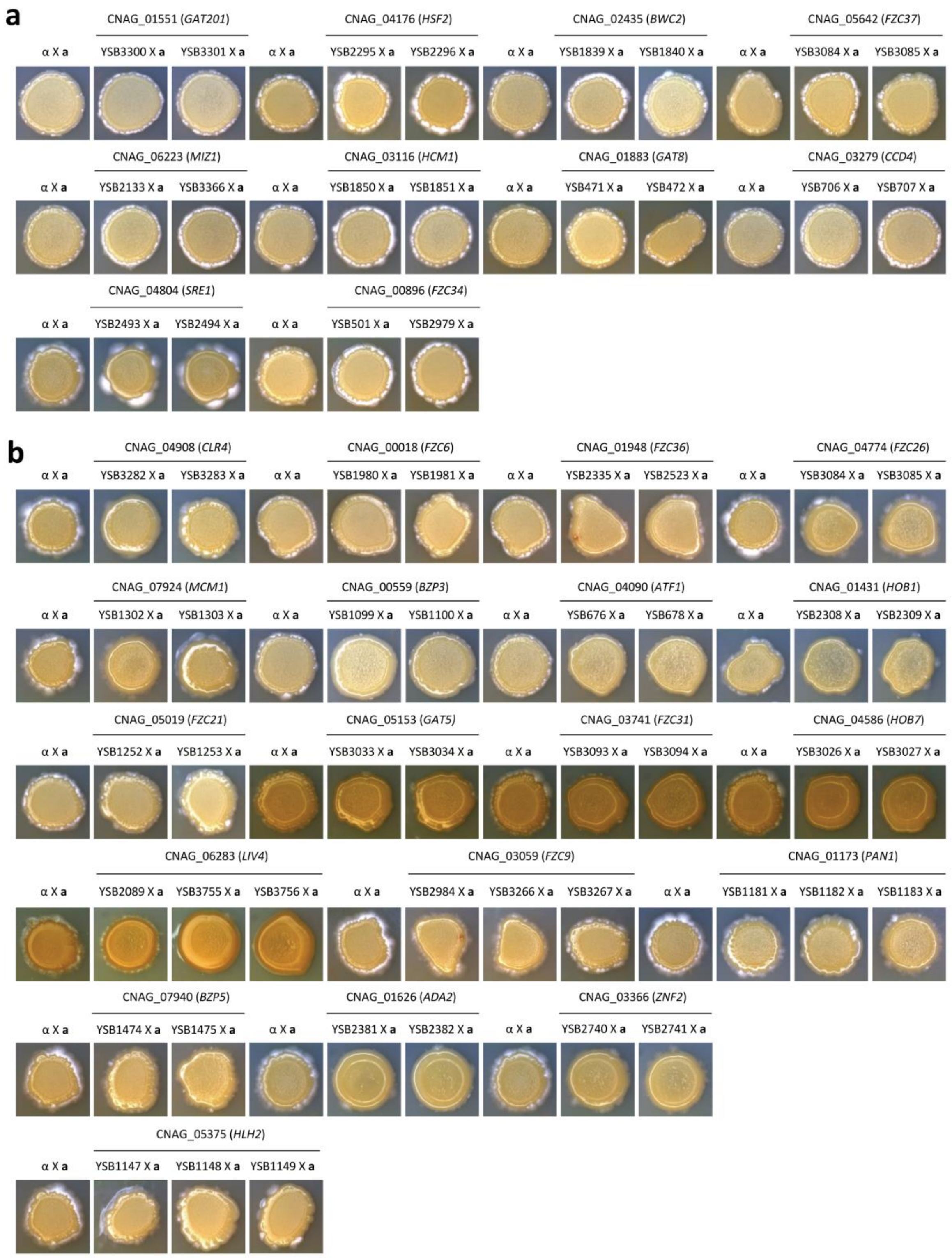

Supplementary Figure 3: TF mutants involved in the sexual differentiation of *C. neoformans*. In the mating assay, the wild-type strain H99 (WT) and each TF mutant were cocultured with the opposite mating type KN99**a** strain on V8 media and incubated at room temperature in the dark for 11 days and then photographed using a microscope (Motic Microscope) equipped with a digital camera (Pro-Microscan No. 5888). (a) and (b) panels include groups of TF mutants showing enhanced and decreased mating efficiency, respectively.

a

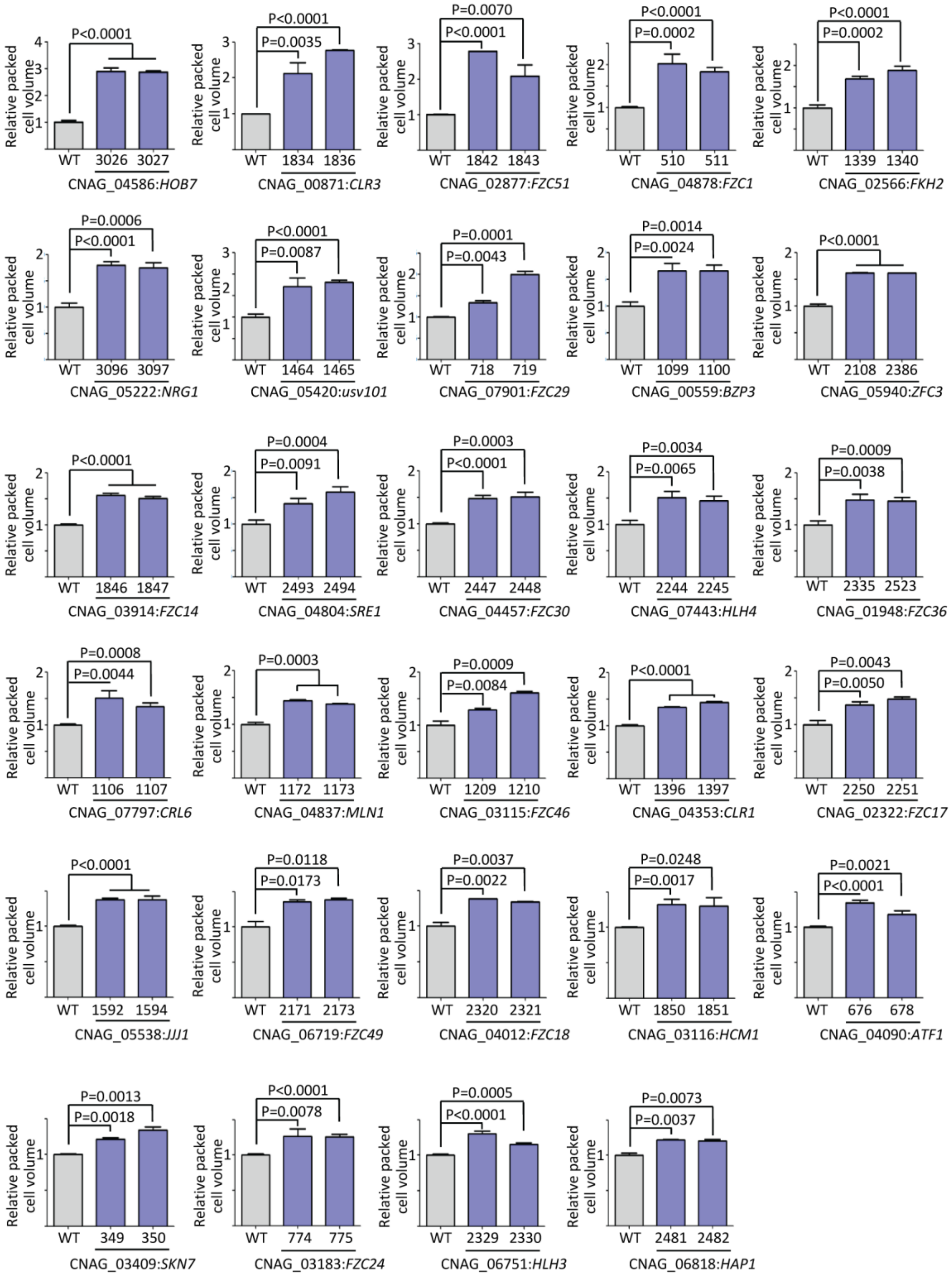
Relative packed cell volume
P<0.0001
WT 3026 3027
CNAG_04586:HOB7
P<0.0001
P=0.0035
WT 1834 1836
CNAG_00871:CLR3
P=0.0070
P<0.0001
WT 1842 1843
CNAG_02877:FZC51
P<0.0001
P=0.0002
WT 510 511
CNAG_04878:FZC1
P<0.0001
P=0.0002
WT 1339 1340
CNAG_02566:FKH2
P=0.0006
P<0.0001
WT 3096 3097
CNAG_05222:NRG1
P<0.0001
P=0.0087
WT 1464 1465
CNAG_05420:usv101
P=0.0001
P=0.0043
WT 718 719
CNAG_07901:FZC29
P=0.0014
P=0.0024
WT 1099 1100
CNAG_00559:BZP3
P<0.0001
WT 2108 2386
CNAG_05940:ZFC3
P<0.0001
WT 1846 1847
CNAG_03914:FZC14
P=0.0004
P=0.0091
WT 2493 2494
CNAG_04804:SRE1
P=0.0003
P<0.0001
WT 2447 2448
CNAG_04457:FZC30
P=0.0034
P=0.0065
WT 2244 2245
CNAG_07443:HLH4
P=0.0009
P=0.0038
WT 2335 2523
CNAG_01948:FZC36
P=0.0008
P=0.0044
WT 1106 1107
CNAG_07797:CRL6
P=0.0003
WT 1172 1173
CNAG_04837:MLN1
P=0.0009
P=0.0084
WT 1209 1210
CNAG_03115:FZC46
P<0.0001
WT 1396 1397
CNAG_04353:CLR1
P=0.0043
P=0.0050
WT 2250 2251
CNAG_02322:FZC17
P<0.0001
WT 1592 1594
CNAG_05538:JJJ1
P=0.0118
P=0.0173
WT 2171 2173
CNAG_06719:FZC49
P=0.0037
P=0.0022
WT 2320 2321
CNAG_04012:FZC18
P=0.0248
P=0.0017
WT 1850 1851
CNAG_03116:HCM1
P=0.0021
P<0.0001
WT 676 678
CNAG_04090:ATF1
P=0.0013
P=0.0018
WT 349 350
CNAG_03409:SKN7
P<0.0001
P=0.0078
WT 774 775
CNAG_03183:FZC24
P=0.0005
P<0.0001
WT 2329 2330
CNAG_06751:HLH3
P=0.0073
P=0.0037
WT 2481 2482
CNAG_06818:HAP1

Continued

b

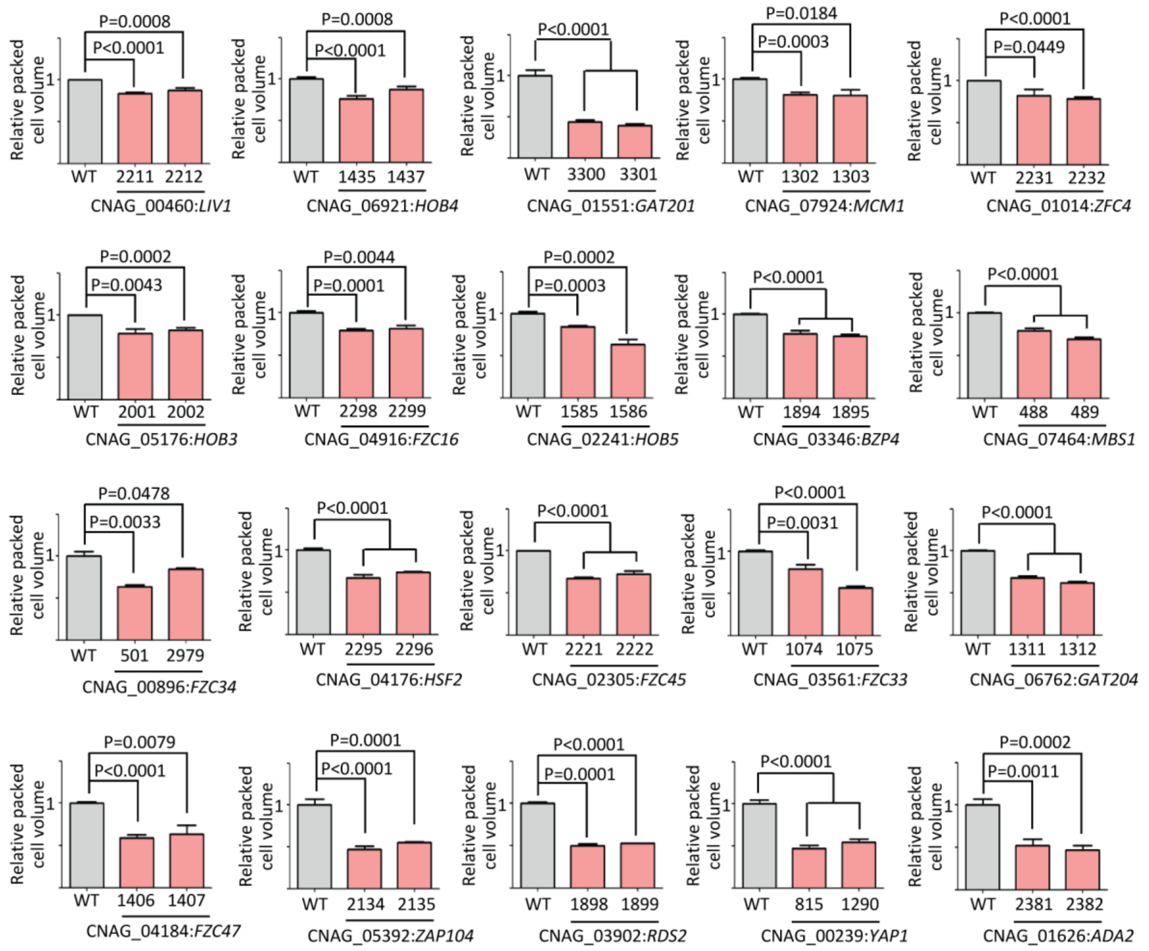

Supplementary Figure 4: TF mutants involved in capsule production in *C. neoformans*. To quantify capsule production, each strain was grown in YPD medium for 16 h at 30°C, spotted on agar-based DME, and incubated at 37°C for 2 days. The cells were then scraped, fixed, and synchronized to equal concentration (5×10^{8} cells ml^{-1}). An equal number of cells (2.5×10^{7} cells ml^{-1}) of each strain was injected into a hematocrit capillary tube and allowed to precipitate by gravity after 10 days. The packed cell volume ratio was measured by calculating the ratio of the length of the packed cell volume phase to the length of the total volume phase (cells + medium) in cryptocrit. The relative packed cell volume of each mutant was measured by calculating the ratio of the mutant packed cell volume ratio to the WT packed cell volume ratio. Three independent technical replicate tests were performed using two independent strains. For statistical tests, one-way ANOVA with Bonferroni's posttest was performed for selected pairs of WT and mutant strains by using Prism 5 (Graph pad software). Two independent TF mutants that are statistically different ($P < 0.05$) are shown in this figure. (a) and (b) panels include groups of TF mutants showing enhanced and decreased capsule production, respectively.

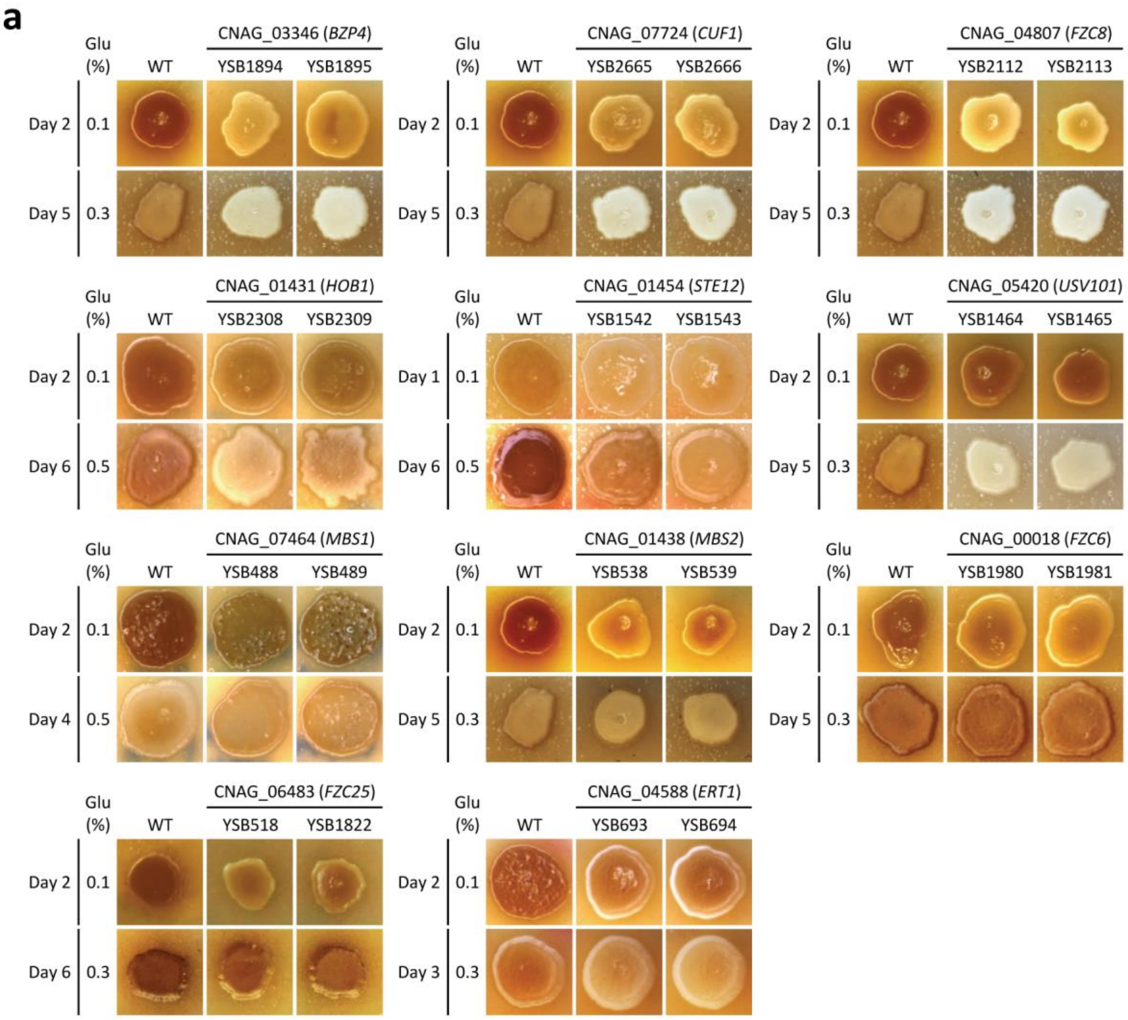
a
Glu (%)
WT
CNAG_03346 (BZP4)
YSB1894
YSB1895
Day 2
0.1
Day 5
0.3
CNAG_07724 (CUF1)
YSB2665
YSB2666
CNAG_04807 (FZC8)
YSB2112
YSB2113
CNAG_01431 (HOB1)
YSB2308
YSB2309
Day 6
0.5
CNAG_01454 (STE12)
YSB1542
YSB1543
Day 1
CNAG_05420 (USV101)
YSB1464
YSB1465
CNAG_07464 (MBS1)
YSB488
YSB489
Day 4
CNAG_01438 (MBS2)
YSB538
YSB539
CNAG_00018 (FZC6)
YSB1980
YSB1981
CNAG_06483 (FZC25)
YSB518
YSB1822
CNAG_04588 (ERT1)
YSB693
YSB694
Day 3

Continued

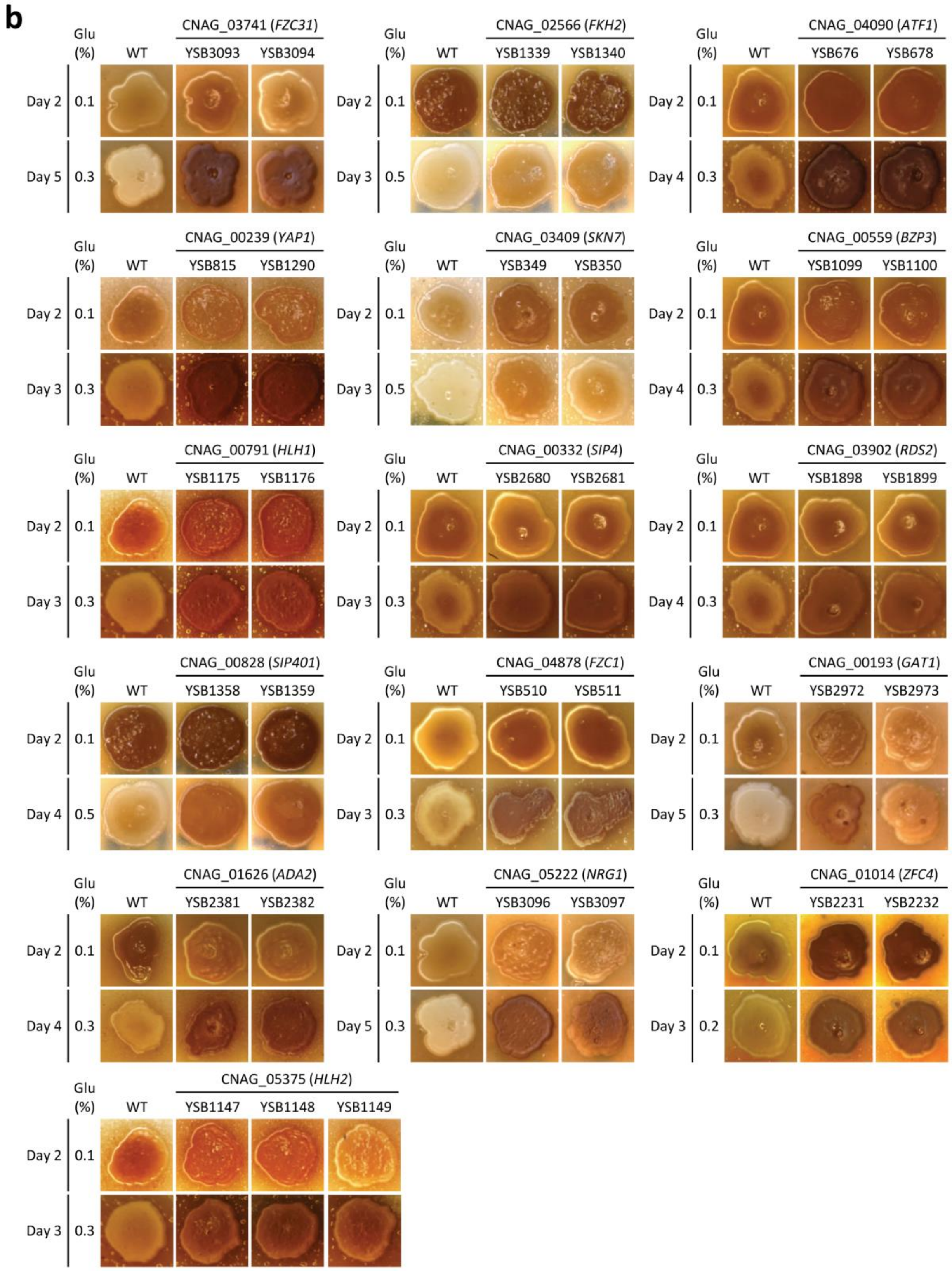

Supplementary Figure 5: TF mutants involved in melanin production in *C. neoformans.* *C. neoformans* strains were cultured overnight in liquid YPD medium at 30°C, after which 5 µl of cultures were spotted on Niger seed agar media containing the indicated concentration of glucose and further incubated at 37°C. (a) and (b) panels include groups of TF mutants showing reduced and enhanced melanin production, respectively

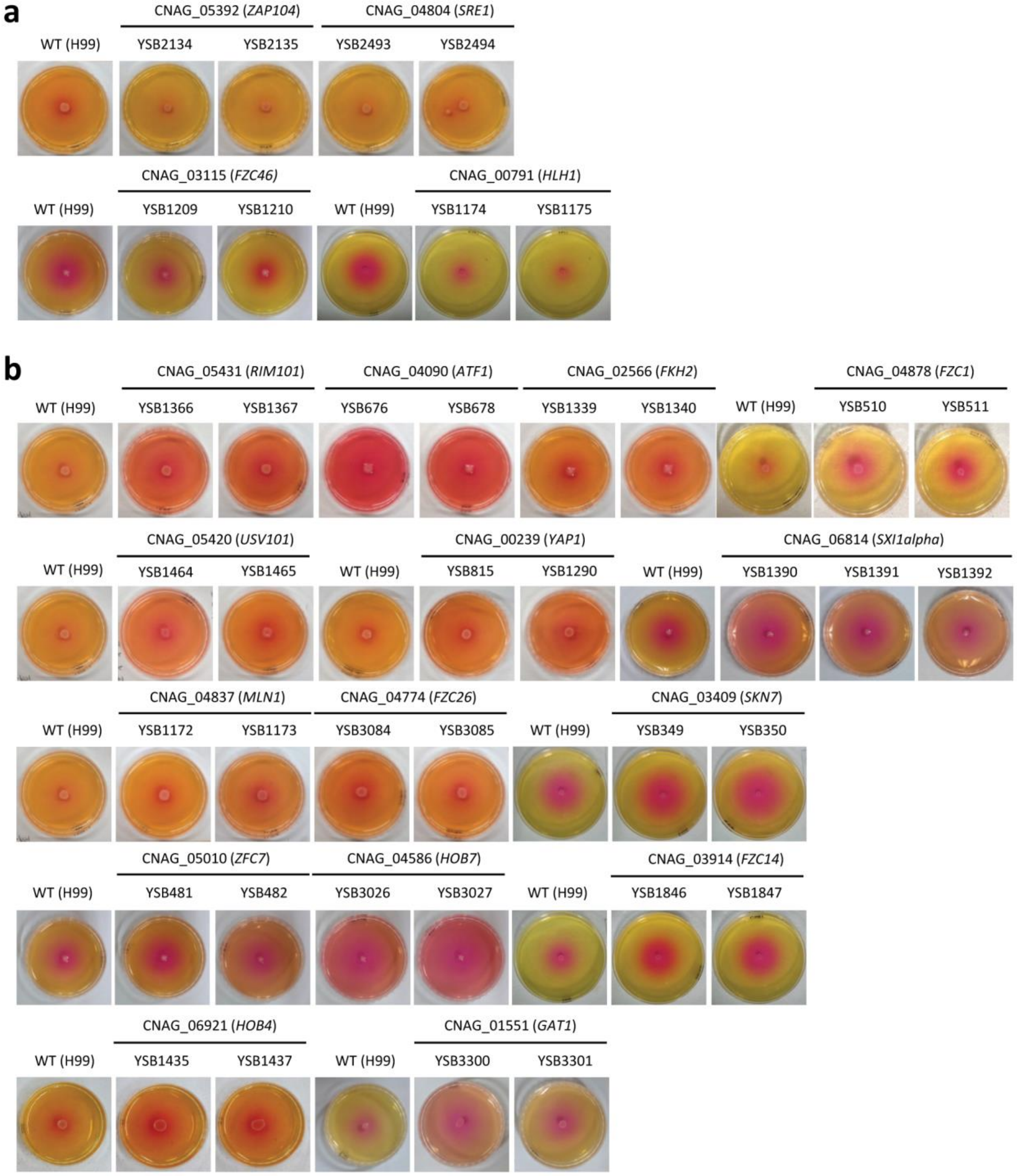

Supplementary Figure 6: TFs required for urease production in *C. neoformans*. *C. neoformans* strains were grown overnight in liquid YPD medium at 30°C. Cells were washed with distilled water and adjusted to 1×10^7 cells ml^{-1} and then 5 µl of cells (5×10^4 cells) were spotted onto Christensen's agar media and further incubated for 7–10 days at 30°C. (a) and (b) panels include groups of TF mutants showing reduced and enhanced urease production, respectively.

a TFs involved in osmotic stress responses

	H99 ID	Designated name	NR	KR	SR	NS	KS	SS
1	04263	*BZP2*						
2	01626	*ADA2*						
3	01431	*HOB1*						
4	07435	*HAP2*						
5	05431	*RIM101*						
6	01948	*FZC36*						
7	00018	*FZC6*						
8	07464	*MBS1*						
9	03409	*SKN7*						
10	05153	*GAT5*						
11	01173	*PAN1*						
12	05170	*PIP2*						
13	00039	*ZFC6*						
14	05380	*FZC44*						
15	03741	*FZC31*						
16	05093	*HOB6*						
17	03116	*HCM1*						
18	04841	*FZC43*						
19	02066	*FZC13*						
20	01708	*GAT7*						
21	07724	*CUF1*						
22	06751	*HLH3*						
23	05222	*NRG1*						
24	04345	*ARO8001*						
25	00068	*MET32*						
26	04630	*YAP2*						
27	03115	*FZC46*						
28	00896	*FZC34*						
29	03527	*HEL2*						
30	05112	*FZC42*						
31	01883	*GAT8*						
32	03346	*BZP4*						
33	06339	*FZC35*						
34	02364	*FZC19*						
35	02877	*FZC51*						
36	03768	*FZC32*						

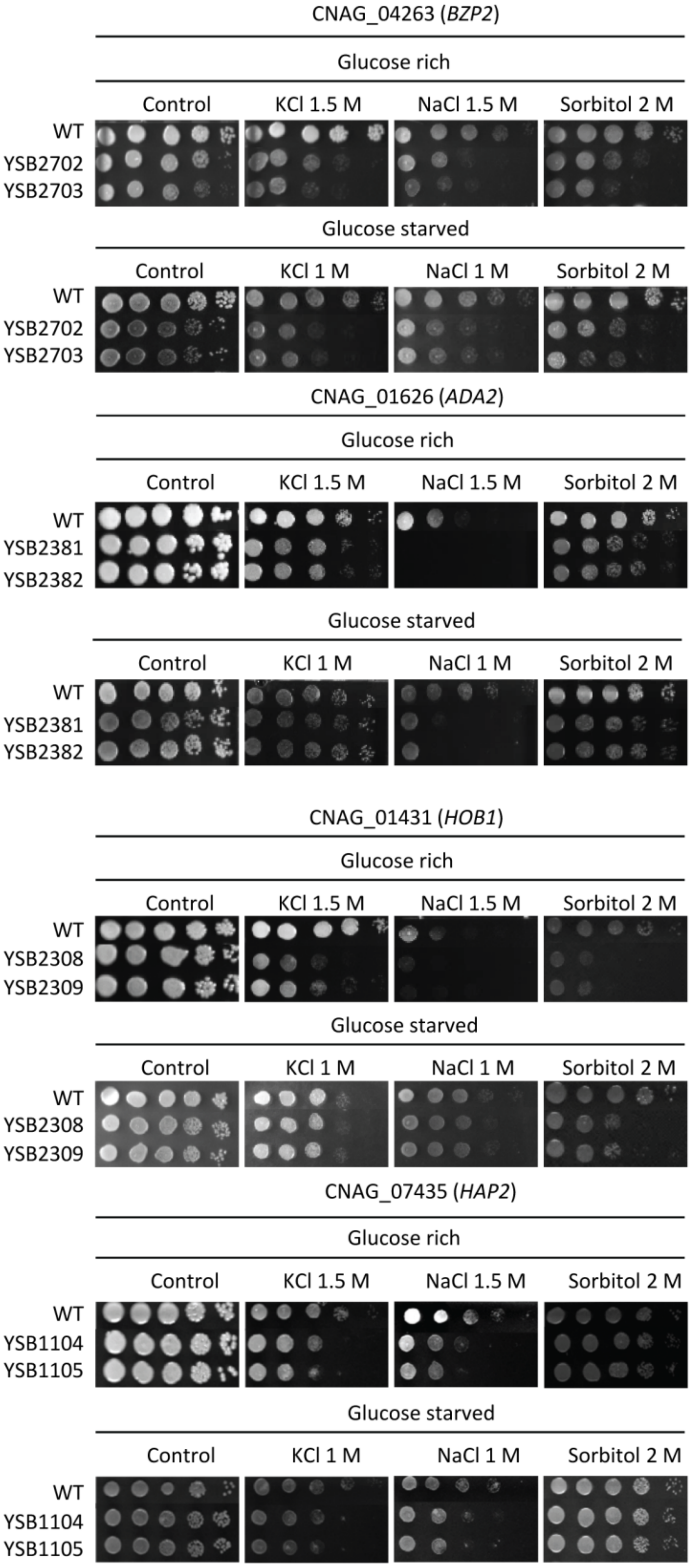

Continued

b TFs involved in oxidative stress responses

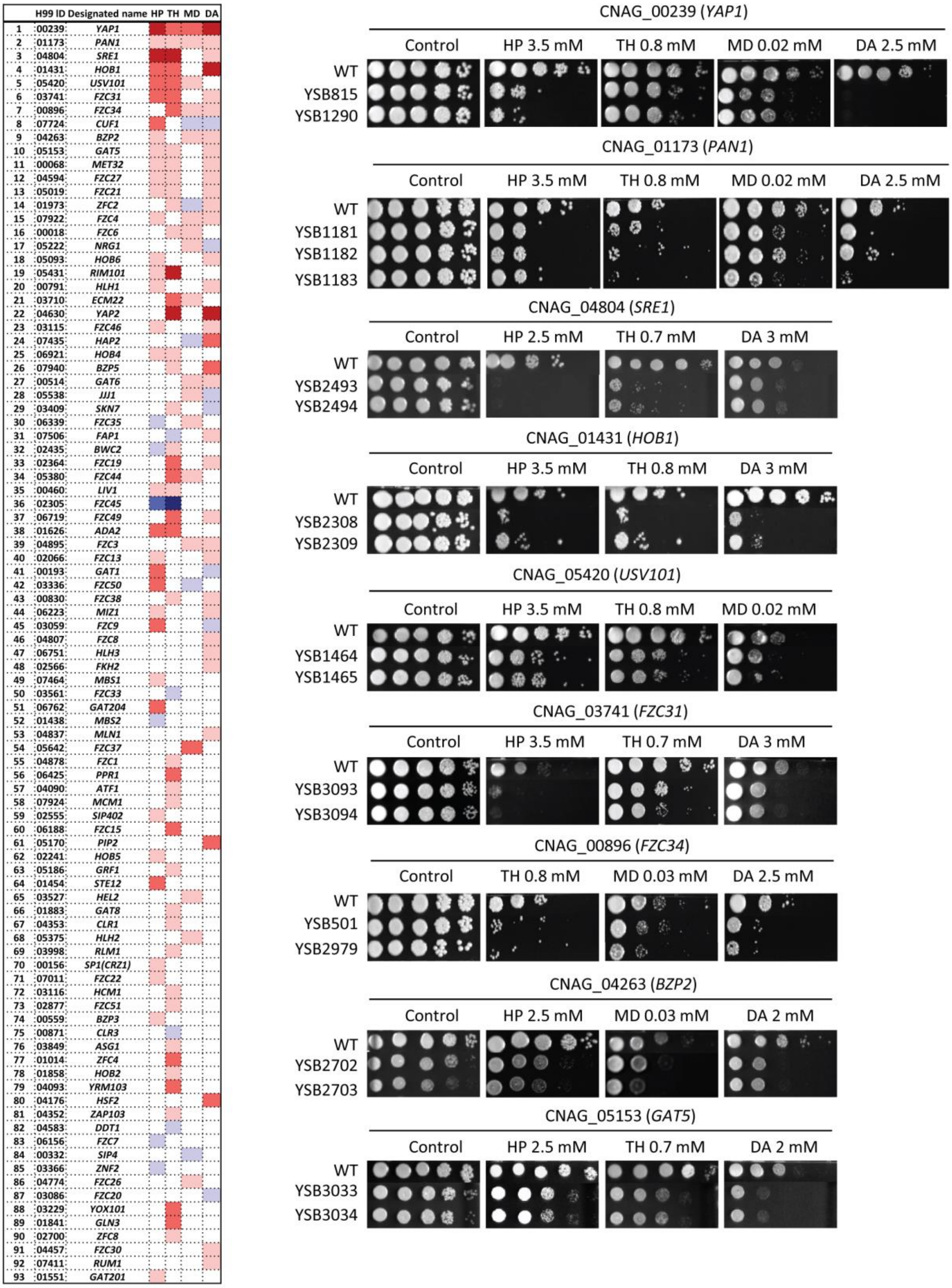

Continued

c TFs involved in ER stress responses

	H99 ID	Designated name	TM	DT
1	06134	*BZP1(HXL1)*		
2	04263	*BZP2*		
3	07724	*CUF1*		
4	01431	*HOB1*		
5	05222	*NRG1*		
6	07464	*MBS1*		
7	07435	*HAP2*		
8	04353	*CLR1*		
9	04804	*SRE1*		
10	05153	*GAT5*		
11	00791	*HLH1*		
12	00068	*MET32*		
13	01173	*PAN1*		
14	05170	*PIP2*		
15	03998	*RLM1*		
16	00559	*BZP3*		
17	05375	*HLH2*		
18	04630	*YAP2*		
19	00018	*FZC6*		
20	06283	*LIV4*		
21	02566	*FKH2*		
22	01069	*FZC11*		
23	03401	*GAT203*		
24	00828	*SIP401*		
25	05785	*STB4*		
26	07593	*YAP4*		
27	04837	*MLN1*		
28	05431	*RIM101*		
29	06425	*PPR1*		
30	01454	*STE12*		
31	03527	*HEL2*		
32	05255	*FZC2*		
33	00239	*YAP1*		
34	00156	*SP1(CRZ1)*		
35	04268	*APN2*		
36	05420	*USV101*		
37	00514	*GAT6*		
38	05538	*JJJ1*		
39	03409	*SKN7*		
40	02435	*BWC2*		
41	03116	*HCM1*		
42	06483	*FZC25*		
43	05019	*FZC21*		
44	05380	*FZC44*		
45	05176	*HOB3*		
46	01014	*ZFC4*		
47	01626	*ADA2*		
48	05940	*ZFC3*		
49	01948	*FZC36*		
50	04583	*DDT1*		
51	01973	*ZFC2*		
52	01708	*GAT7*		
53	03902	*RDS2*		
54	00193	*GAT1*		
55	03086	*FZC20*		
56	02700	*ZFC8*		
57	04586	*HOB7*		
58	03741	*FZC31*		
59	01551	*GAT201*		
60	04908	*CLR4*		

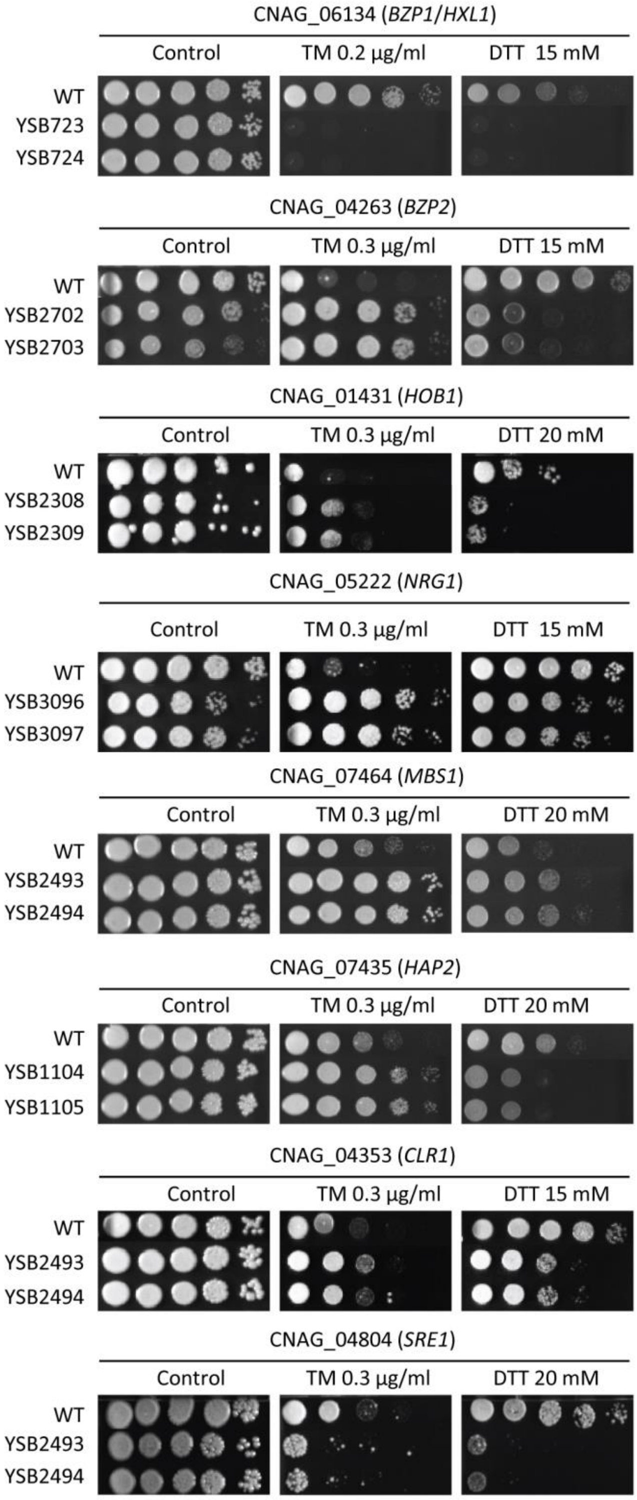

Continued

d TFs involved in genotoxic stress responses

	H99 ID	Designated name	MS	HU
1	01431	*HOB1*		
2	00514	*GAT6*		
3	04804	*SRE1*		
4	05153	*GAT5*		
5	06134	*BZP1(HXL1)*		
6	00018	*FZC6*		
7	04263	*BZP2*		
8	05538	*JJJ1*		
9	06223	*MIZ1*		
10	05222	*NRG1*		
11	07464	*MBS1*		
12	04878	*FZC1*		
13	05170	*PIP2*		
14	05375	*HLH2*		
15	04268	*APN2*		
16	03409	*SKN7*		
17	03116	*HCM1*		
18	03212	*HCM101*		
19	01626	*ADA2*		
20	07922	*FZC4*		
21	03086	*FZC20*		
22	03229	*YOX101*		

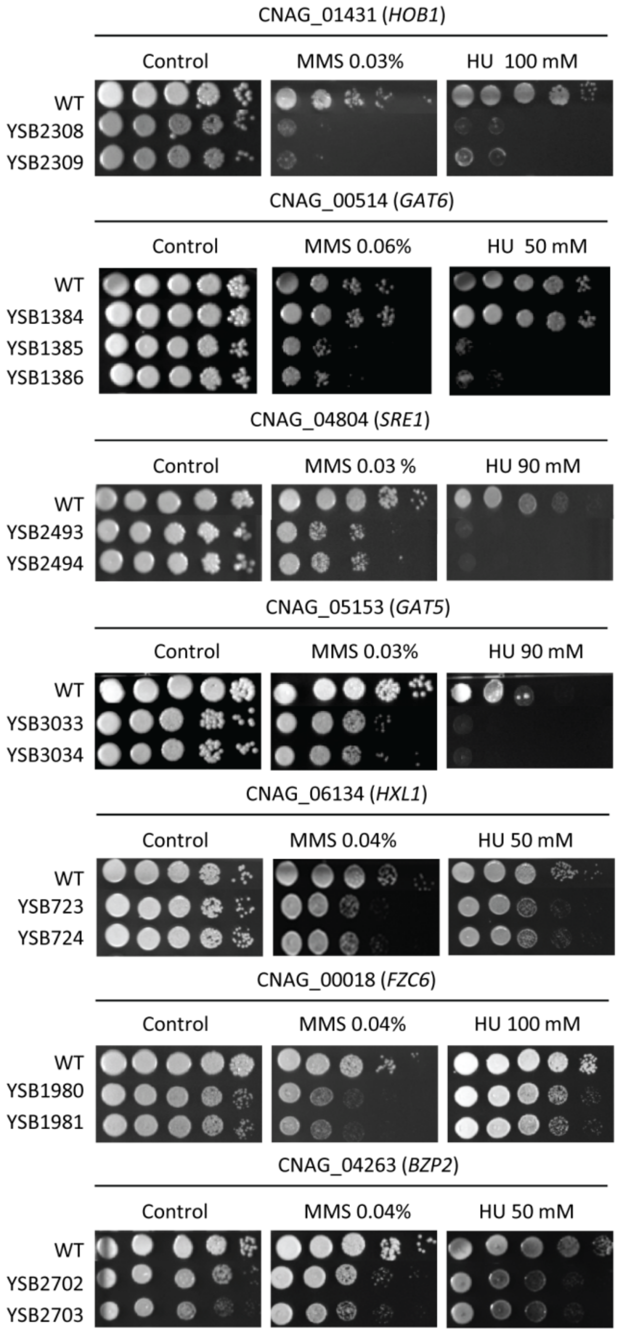

Continued

e TFs involved in cell wall/membrane stress responses

	H99 ID	Designated name	CW	CR	SD
1	00156	*SP1(CRZ1)*			
2	01431	*HOB1*			
3	06751	*HLH3*			
4	05222	*NRG1*			
5	07435	*HAP2*			
6	04263	*BZP2*			
7	06134	*BZP1(HXL1)*			
8	07724	*CUF1*			
9	05431	*RIM101*			
10	04630	*YAP2*			
11	00018	*FZC6*			
12	05153	*GAT5*			
13	04588	*ERT1*			
14	04878	*FZC1*			
15	03710	*ECM22*			
16	01173	*PAN1*			
17	06188	*FZC15*			
18	05170	*PIP2*			
19	02241	*HOB5*			
20	05186	*GRF1*			
21	00039	*ZFC6*			
22	07940	*BZP5*			
23	06814	*SXI1alpha*			
24	04353	*CLR1*			
25	03998	*RLM1*			
26	00239	*YAP1*			
27	05420	*USV101*			
28	00514	*GAT6*			
29	05538	*JJJ1*			
30	03409	*SKN7*			
31	07011	*FZC22*			
32	07506	*FAP1*			
33	04807	*FZC8*			
34	02435	*BWC2*			
35	02877	*FZC51*			
36	00559	*BZP3*			
37	07797	*CRL6*			
38	05019	*FZC21*			
39	05176	*HOB3*			
40	03849	*ASG1*			
41	01014	*ZFC4*			
42	04804	*SRE1*			
43	02603	*ZFC1*			
44	04036	*HSF3*			
45	06156	*FZC7*			
46	03018	*ASG101*			
47	01708	*GAT7*			
48	00332	*SIP4*			
49	03902	*RDS2*			
50	04774	*FZC26*			
51	03059	*FZC9*			
52	00193	*GAT1*			
53	03336	*FZC50*			
54	02723	*FZC23*			
55	03741	*FZC31*			
56	04457	*FZC30*			
57	07411	*RUM1*			
58	01551	*GAT201*			
59	04908	*CLR4*			

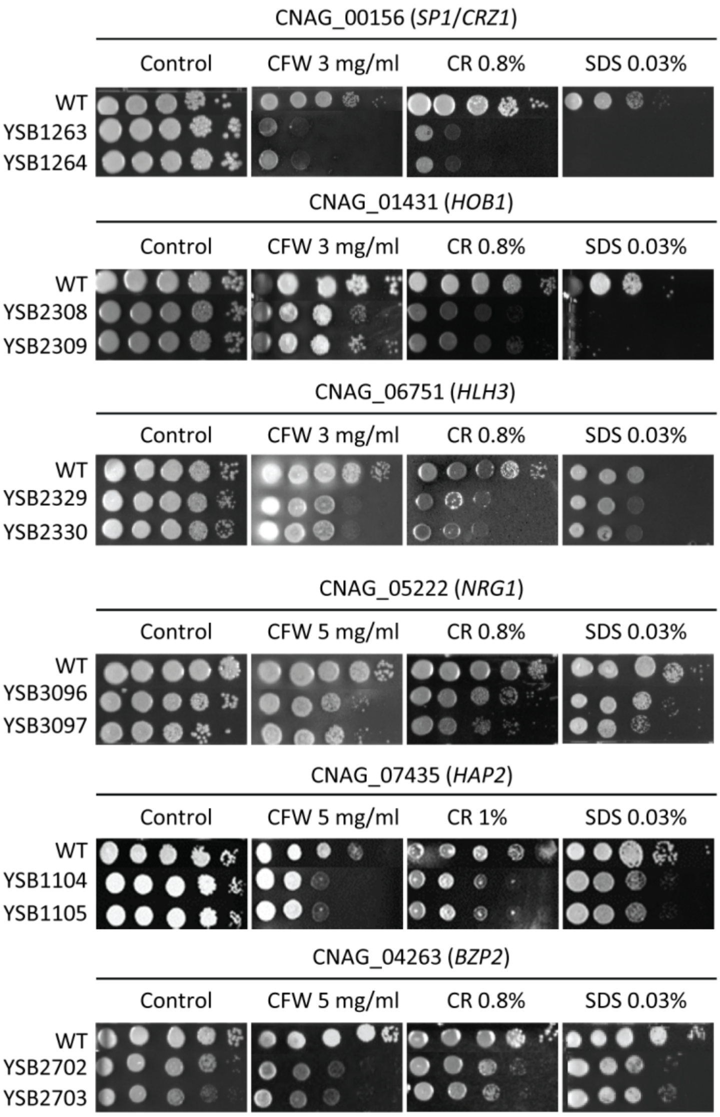

Continued

f TFs involved in heavy metal stress responses

	H99 ID	Designated name	CD
1	07724	*CUF1*	
2	00018	*FZC6*	
3	05153	*GAT5*	
4	05642	*FZC37*	
5	07435	*HAP2*	
6	03409	*SKN7*	
7	04263	*BZP2*	
8	01438	*MBS2*	
9	04837	*MLN1*	
10	05093	*HOB6*	
11	04398	*ARO80*	
12	03279	*CCD4*	
13	04345	*ARO8001*	
14	04184	*FZC47*	
15	03115	*FZC46*	
16	05170	*PIP2*	
17	02241	*HOB5*	
18	00239	*YAP1*	
19	03346	*BZP4*	
20	06339	*FZC35*	
21	07506	*FAP1*	
22	04807	*FZC8*	
23	02364	*FZC19*	
24	03116	*HCM1*	
25	02877	*FZC51*	
26	05049	*PIP201*	
27	03229	*YOX101*	
28	01841	*GLN3*	
29	02700	*ZFC8*	
30	04586	*HOB7*	
31	07411	*RUM1*	
32	04836	*FZC10*	
33	05222	*NRG1*	
34	06762	*GAT204*	
35	04090	*ATF1*	
36	04353	*CLR1*	
37	05375	*HLH2*	
38	03998	*RLM1*	
39	01626	*ADA2*	
40	05940	*ZFC3*	
41	04804	*SRE1*	
42	01977	*FZC39*	
43	04036	*HSF3*	
44	06156	*FZC7*	
45	03018	*ASG101*	
46	01708	*GAT7*	
47	03086	*FZC20*	
48	00332	*SIP4*	
49	03902	*RDS2*	
50	03366	*ZNF2*	
51	03336	*FZC50*	
52	01551	*GAT201*	
53	06134	*BZP1(HXL1)*	

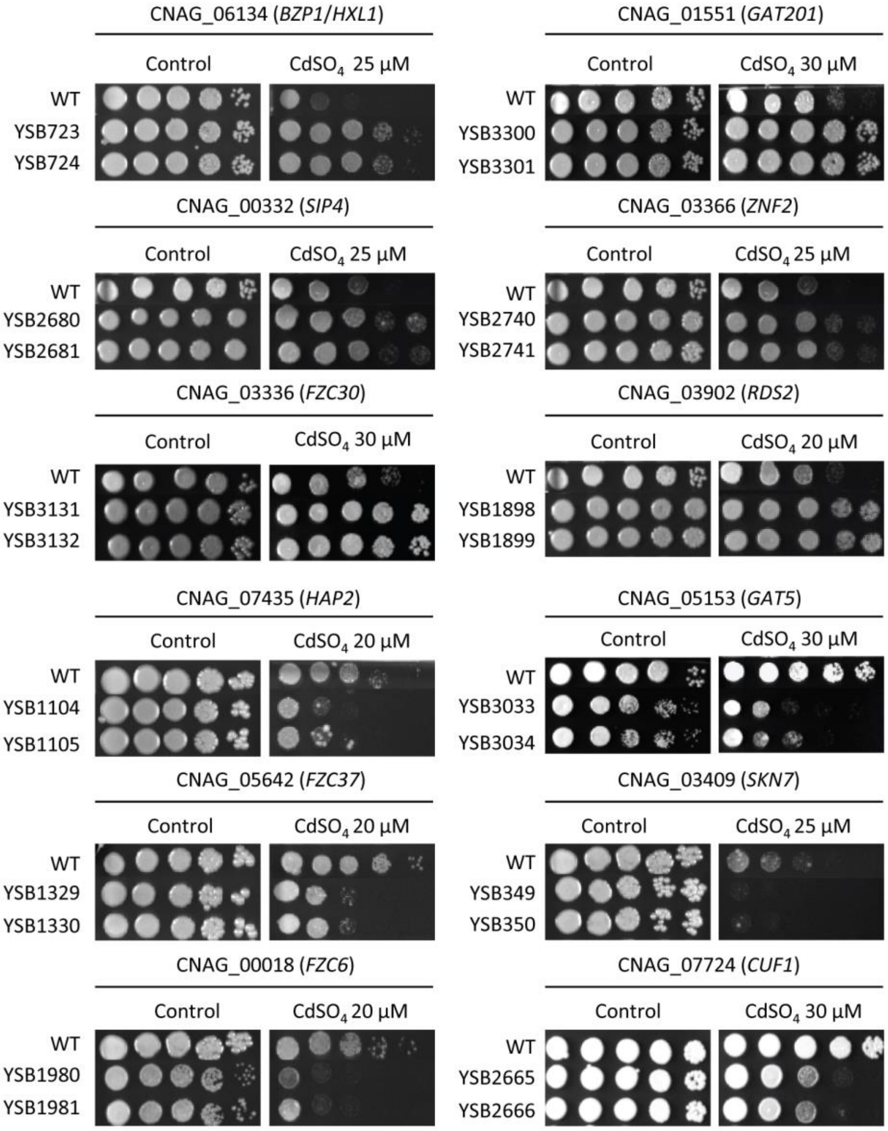

Supplementary Figure 7. Selected list of TFs involved in stress response and adaption of *C. neoformans*. *C. neoformans* strains were cultured for 16 h in liquid medium at 30°C. Cells were washed with distilled water and 10-fold serially diluted (1 to 10^4 dilutions) and then spotted (3 µl of each dilution) onto YPD containing the indicated concentrations of stress inducing reagents. a: Osmotic stress; b: Oxidative stress (HP (H_2O_2), TH (*tert*-butyl hydroperoxide), MD (menadione), DA (diamide)); c: ER stress (TM (tunicamycin), DTT (dithiothreitol)); d: Genotoxic stress (MMS (methyl methanesulfonate), HU (hydroxyurea)); e: Cell wall/membrane stress (CFW (calcofluor white), CR (Congo red), SDS (sodium dodecyl sulfate)); and f: Heavy metal stress. In the left side of each panel, the phenome heat map for each stress response was indicated.

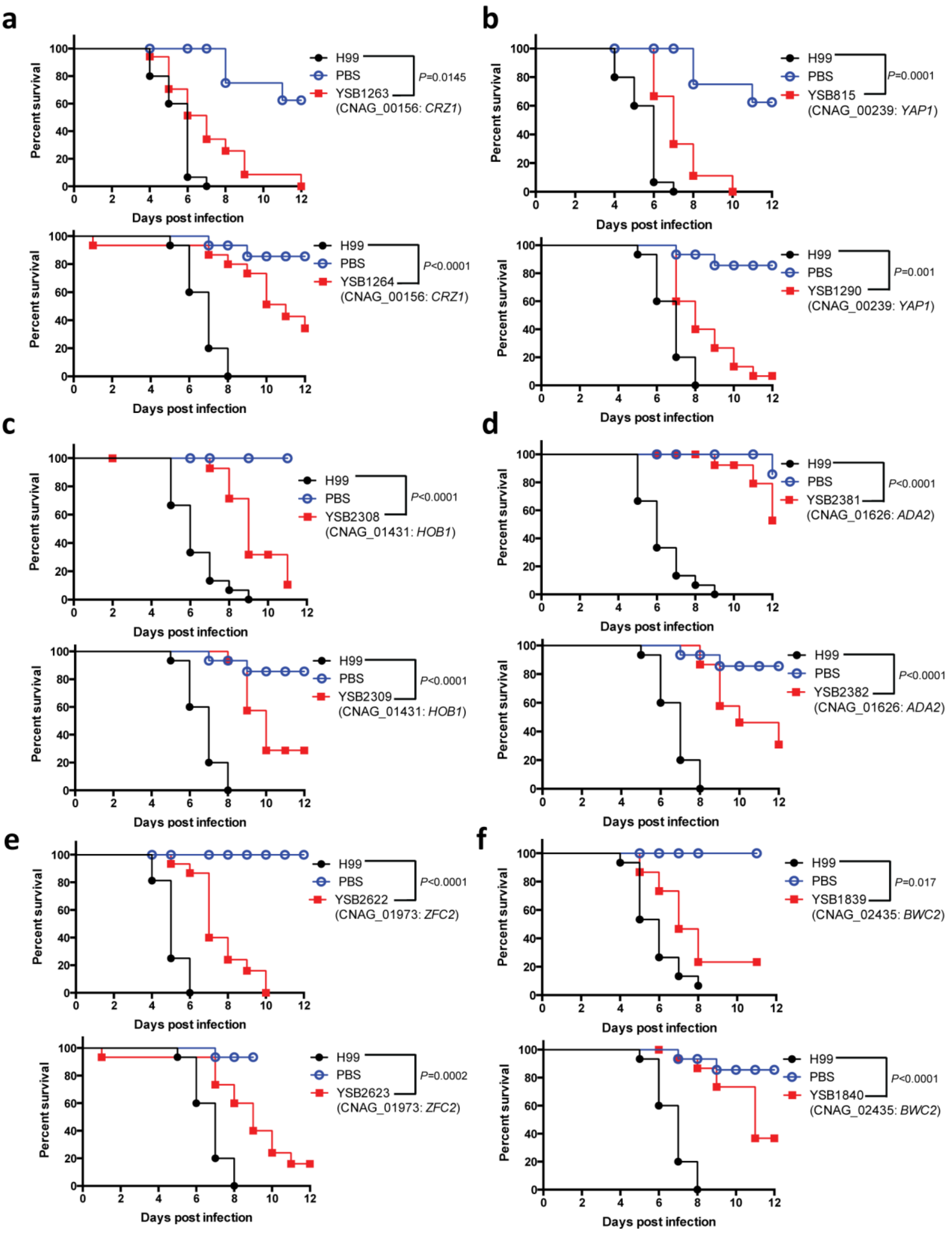
a
Percent survival
Days post infection
H99
PBS
YSB1263
(CNAG_00156: CRZ1)
P=0.0145
YSB1264
(CNAG_00156: CRZ1)
P<0.0001
b
YSB815
(CNAG_00239: YAP1)
P=0.0001
YSB1290
(CNAG_00239: YAP1)
P=0.001
c
YSB2308
(CNAG_01431: HOB1)
P<0.0001
YSB2309
(CNAG_01431: HOB1)
P<0.0001
d
YSB2381
(CNAG_01626: ADA2)
P<0.0001
YSB2382
(CNAG_01626: ADA2)
P<0.0001
e
YSB2622
(CNAG_01973: ZFC2)
P<0.0001
YSB2623
(CNAG_01973: ZFC2)
P=0.0002
f
YSB1839
(CNAG_02435: BWC2)
P=0.017
YSB1840
(CNAG_02435: BWC2)
P<0.0001

Continued

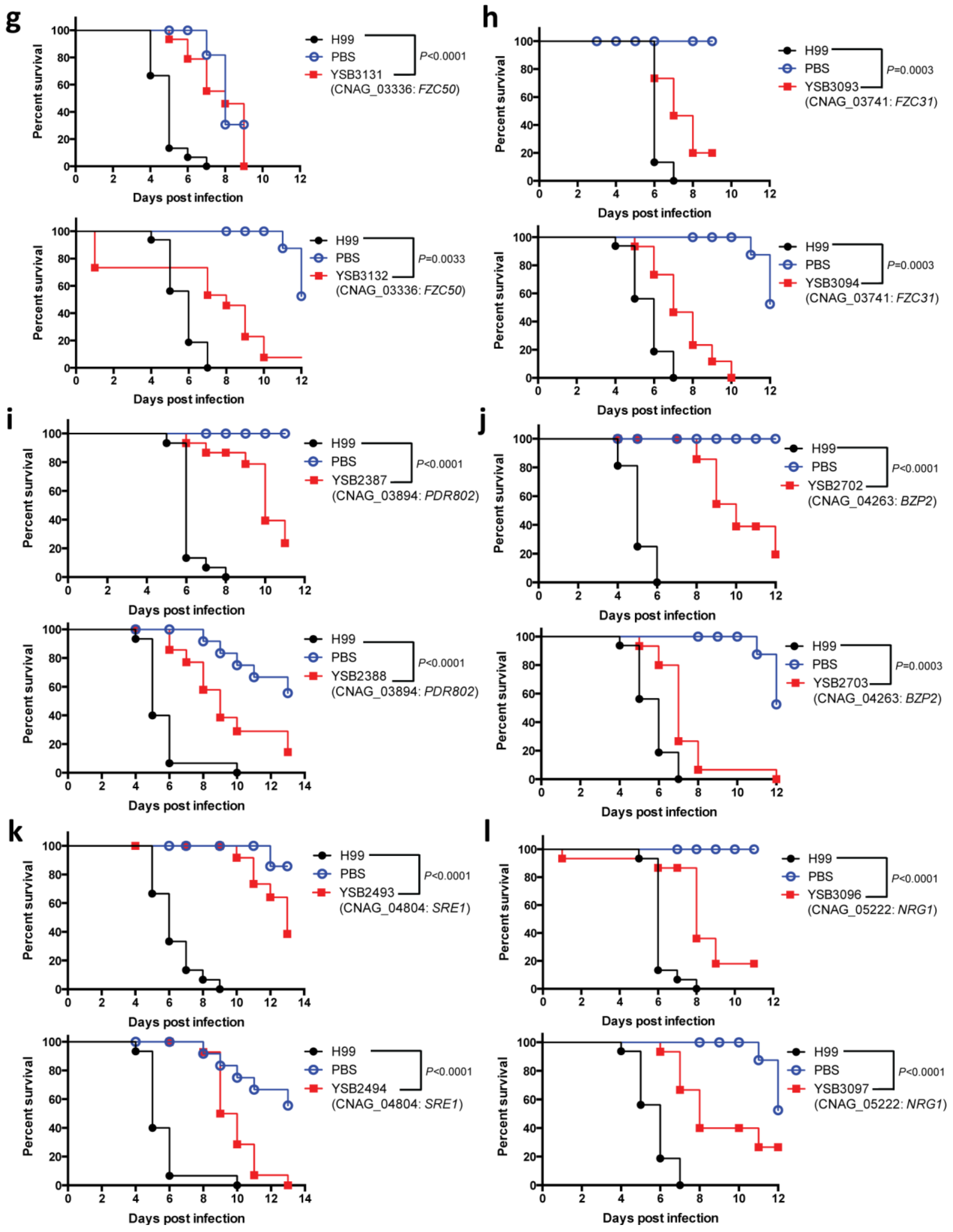
g
H99
PBS
YSB3131
(CNAG_03336: FZC50)
P<0.0001
YSB3132
(CNAG_03336: FZC50)
P=0.0033
h
YSB3093
(CNAG_03741: FZC31)
P=0.0003
YSB3094
(CNAG_03741: FZC31)
P=0.0003
i
YSB2387
(CNAG_03894: PDR802)
P<0.0001
YSB2388
(CNAG_03894: PDR802)
P<0.0001
j
YSB2702
(CNAG_04263: BZP2)
P<0.0001
YSB2703
(CNAG_04263: BZP2)
P=0.0003
k
YSB2493
(CNAG_04804: SRE1)
P<0.0001
YSB2494
(CNAG_04804: SRE1)
P<0.0001
l
YSB3096
(CNAG_05222: NRG1)
P<0.0001
YSB3097
(CNAG_05222: NRG1)
P<0.0001
Percent survival
Days post infection

Continued

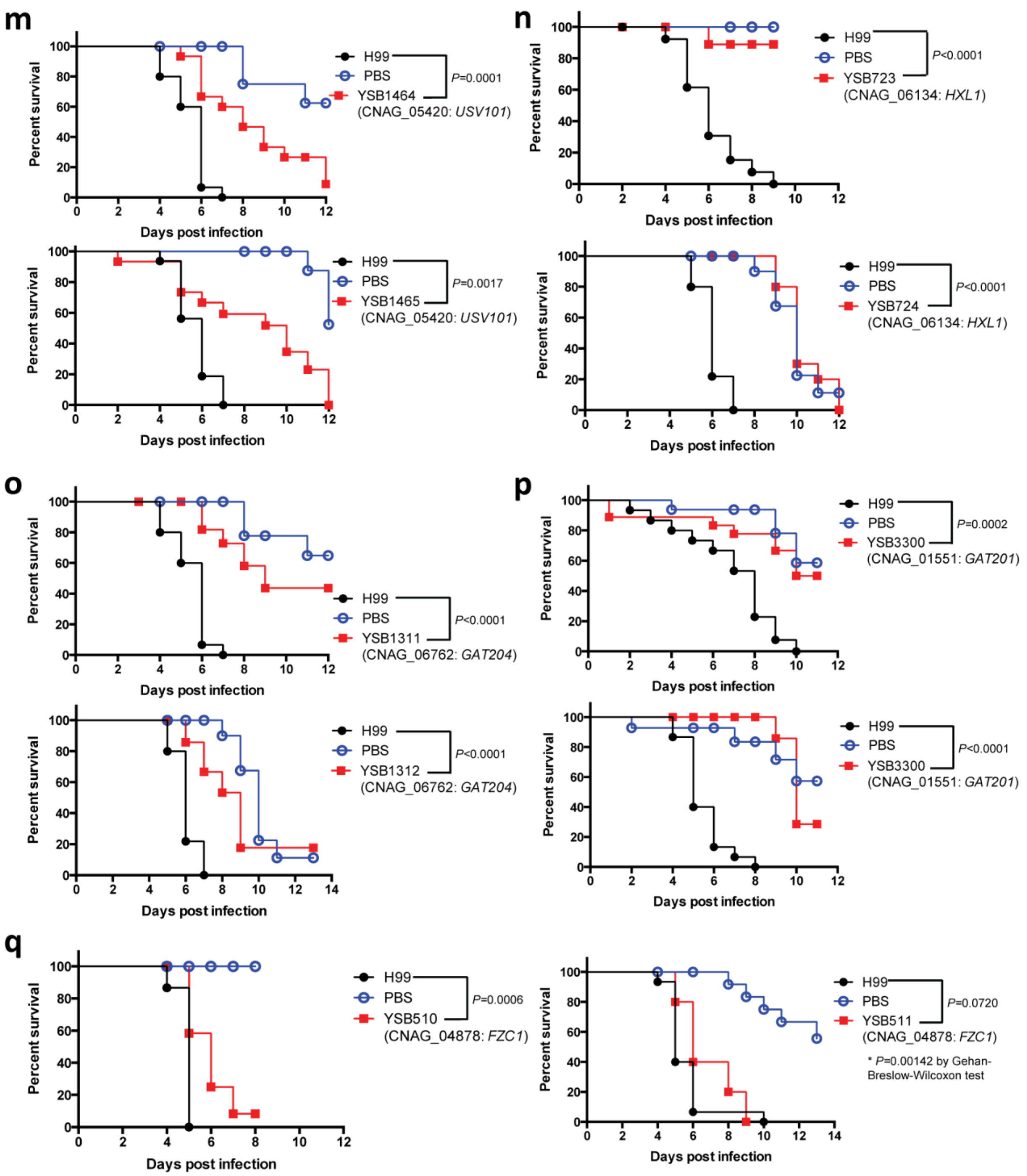

Supplementary Figure 8: Identification of TFs involved in the virulence of *C. neoformans* by using the *Galleria mellonella* killing assay. *C. neoformans* strains were grown overnight in liquid YPD medium, washed three times with phosphate-buffered saline (PBS), and inoculated into *G. mellonella* at the last larval stage at 4,000 cells/larva (15 larvae per group). The infected larvae were incubated at 37°C and their survival patterns were monitored up to 14 days. Statistical analysis was performed using the Log-rank (Mantel-Cox) test. Each panel (a to l) includes the survival data for two independent mutants for each TF.

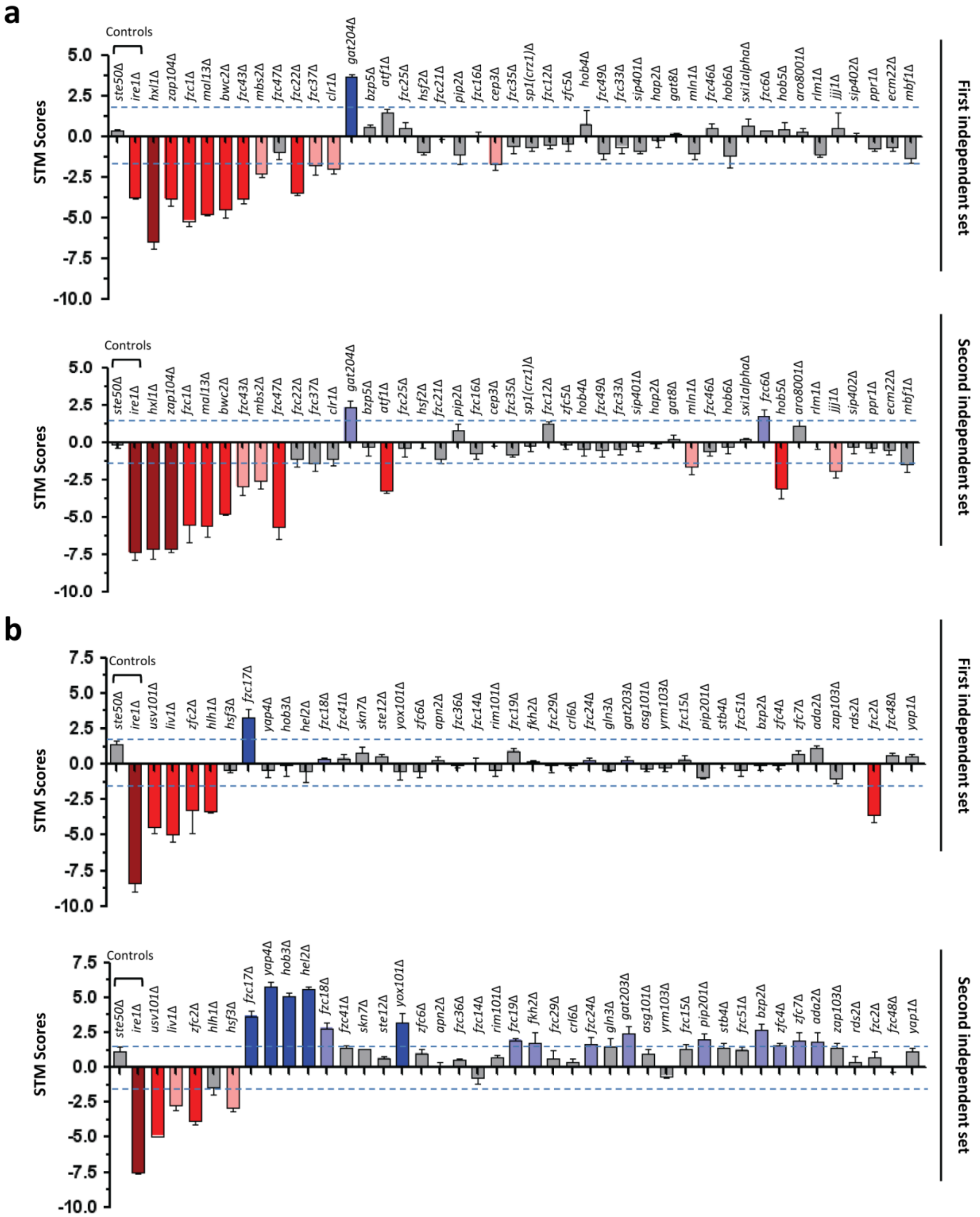
a
Controls
STM Scores
5.0
2.5
0.0
-2.5
-5.0
-7.5
-10.0
ste50Δ ire1Δ hxl1Δ zap104Δ fzc1Δ mal13Δ bwc2Δ fzc43Δ mbs2Δ fzc47Δ fzc22Δ fzc37Δ clr1Δ gat204Δ bzp5Δ atf1Δ fzc25Δ hsf2Δ fzc21Δ pip2Δ fzc16Δ cep3Δ fzc35Δ sp1(crz1)Δ fzc12Δ zfc5Δ hob4Δ fzc49Δ fzc33Δ sip401Δ hap2Δ gat8Δ mln1Δ fzc46Δ hob6Δ sxi1alphaΔ fzc6Δ hob5Δ aro8001Δ rlm1Δ jjj1Δ sip402Δ ppr1Δ ecm22Δ mbf1Δ
First independent set
Controls
STM Scores
Second independent set
b
Controls
STM Scores
7.5
5.0
2.5
0.0
-2.5
-5.0
-7.5
-10.0
ste50Δ ire1Δ usv101Δ liv1Δ zfc2Δ hlh1Δ hsf3Δ fzc17Δ yap4Δ hob3Δ hel2Δ fzc18Δ fzc41Δ skn7Δ ste12Δ yox101Δ zfc6Δ apn2Δ fzc36Δ fzc14Δ rim101Δ fzc19Δ fkh2Δ fzc29Δ crl6Δ fzc24Δ gln3Δ gat203Δ asg101Δ yrm103Δ fzc15Δ pip201Δ stb4Δ fzc51Δ bzp2Δ zfc4Δ zfc7Δ ada2Δ zap103Δ rds2Δ fzc2Δ fzc48Δ yap1Δ
First independent set
Controls
STM Scores
Second independent set

Continued

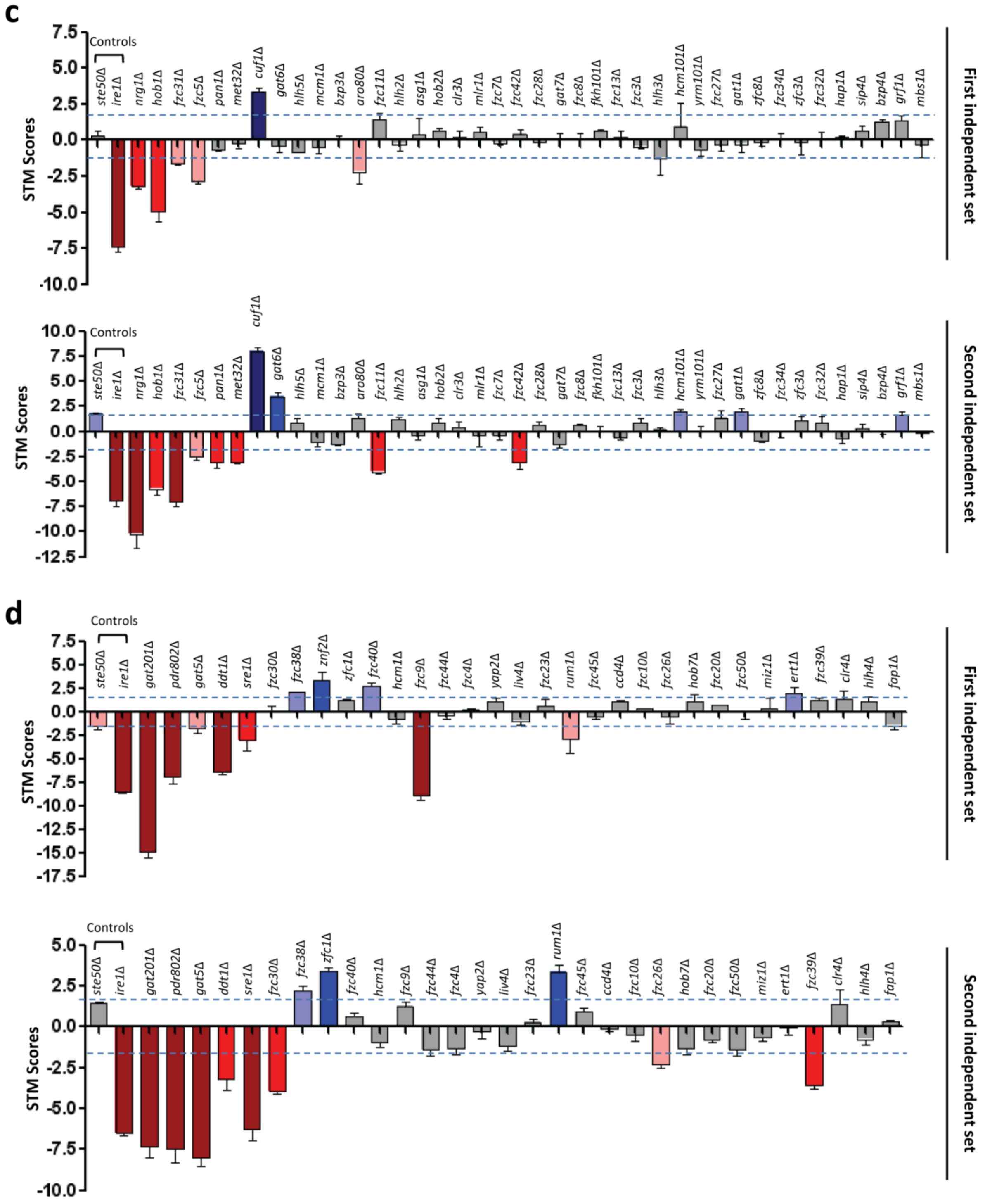

Supplementary Figure 9: Signature-tag mutagenesis (STM) scores of the *C. neoformans* TF mutants. (a–d) STM scores of each TF mutant were measured by means of quantitative PCR analysis performed using a common primer and the signature tag-specific primers listed in Supplemental Table S1. Blue-dotted lines indicate STM score cutoffs (±1.5) above which we consider the change to be a meaningful STM change. The cutoff value was set based on the range of STM scores of the *ste50*Δ mutant, which is the virulent control strain. [Strain information: (a) set1: *hxl1*Δ (YSB723), *zap104*Δ (YSB2134), *fzc1*Δ (YSB510), *mal13*Δ (YSB506), *bwc2*Δ (YSB1839), *fzc43*Δ (YSB517), *mbs2*Δ (YSB538), *fzc47*Δ (YSB1406), *fzc22*Δ (YSB1688), *fzc37*Δ (YSB1329), *clr1*Δ

(YSB1396), *gat204*Δ (YSB1311), *bzp5*Δ (YSB1474), *atf1*Δ (YSB676), *fzc25*Δ (YSB518), *hsf2*Δ (YSB2295), *fzc21*Δ (YSB1252), *pip2*Δ (YSB1249), *fzc16*Δ (YSB2326), *cep3*Δ (YSB847), *fzc35*Δ (YSB1341), *sp1*Δ/*crz1*Δ (YSB1263), *fzc12*Δ (YSB467), *zfc5*Δ (YSB2177), *hob4*Δ (YSB1435), *fzc49*Δ (YSB2171), *fzc33*Δ (YSB1074), *sip401*Δ (YSB1358), *hap2*Δ (YSB1104), *gat8*Δ (YSB471), *mln1*Δ (YSB1172), *fzc46*Δ (YSB1209), *hob6*Δ (YSB1255), *sxi1alpha*Δ (YSB1390), *fzc6*Δ (YSB1980), *hob5*Δ (YSB1586), *aro8001*Δ (YSB661), *rlm1*Δ (YSB1300), *jjj1*Δ (YSB1592), *sip402*Δ (YSB529), *ppr1*Δ (YSB1046), *ecm22*Δ (YSB476), *mbf1*Δ (YSB768). set2: *hxl1*Δ (YSB724), *zap104*Δ (YSB2135), *fzc1*Δ (YSB511), *mal13*Δ (YSB507), *bwc2*Δ (YSB1840), *fzc43*Δ (YSB2334), *mbs2*Δ (YSB539), *fzc47*Δ (YSB1407), *fzc22*Δ (YSB1689), *fzc37*Δ (YSB1330), *clr1*Δ (YSB1397), *gat204*Δ (YSB1312), *bzp5*Δ (YSB1475), *atf1*Δ (YSB678), *fzc25*Δ (YSB1822), *hsf2*Δ (YSB2296), *fzc21*Δ (YSB1253), *pip2*Δ (YSB1250), *fzc16*Δ (YSB2327), *cep3*Δ (YSB848), *fzc35*Δ (YSB1342), *sp1*Δ/*crz1*Δ (YSB1264), *fzc12*Δ (YSB468), *zfc5*Δ (YSB2178), *hob4*Δ (YSB1437), *fzc49*Δ (YSB2173), *fzc33*Δ (YSB1075), *sip401*Δ (YSB1359), *hap2*Δ (YSB1105), *gat8*Δ (YSB472), *mln1*Δ (YSB1173), *fzc46*Δ (YSB1210), *hob6*Δ (YSB1256), *sxi1alpha*Δ (YSB1391), *fzc6*Δ (YSB1981), *hob5*Δ (YSB1585), *aro8001*Δ (YSB662), *rlm1*Δ (YSB1301), *jjj1*Δ (YSB1594), *sip402*Δ (YSB530), *ppr1*Δ (YSB1047), *ecm22*Δ (YSB478), *mbf1*Δ (YSB769). (b) set1: *usv101*Δ (YSB1464), *liv1*Δ (YSB2211), *zfc2*Δ (YSB2622), *hlh1*Δ (YSB1175), *hsf3*Δ (YSB2527), *fzc17*Δ (YSB2250), *yap4*Δ (YSB1587), *hob3*Δ (YSB2001), *hel2*Δ (YSB1382), *fzc18*Δ (YSB2320), *skn7*Δ (YSB349), *ste12*Δ (YSB1542), *yox101*Δ (YSB3134), *zfc6*Δ (YSB1953), *apn2*Δ (YSB1429), *fzc36*Δ (YSB2335), *fzc14*Δ (YSB1846), *rim101*Δ (YSB1366), *fzc19*Δ (YSB2115), *fkh2*Δ (YSB1339), *fzc29*Δ (YSB718), *crl6*Δ (YSB1106), *fzc24*Δ (YSB774), *gln3*Δ (YSB3154), *gat203*Δ (YSB569), *asg101*Δ (YSB2697), *yrm103*Δ (YSB2298), *fzc15*Δ (YSB646), *pip201*Δ (YSB3099), *stb4*Δ (YSB1013), *fzc51*Δ (YSB1842), *bzp2*Δ (YSB2702), *zfc4*Δ (YSB2231), *zfc7*Δ (YSB481), *ada2*Δ (YSB2381), *zap103*Δ (YSB2540), *rds2*Δ (YSB18989), *fzc2*Δ (YSB1050), *fzc48*Δ (YSB2646), and *yap1*Δ (YSB815). set2: *usv101*Δ (YSB1465), *liv1*Δ (YSB2212), *zfc2*Δ (YSB2623), *hlh1*Δ (YSB1176), *hsf3*Δ (YSB2528), *fzc17*Δ (YSB2251), *yap4*Δ (YSB1661), *hob3*Δ (YSB2002), *hel2*Δ (YSB1383), *fzc18*Δ (YSB2321), *skn7*Δ (YSB350), *ste12*Δ (YSB1543), *yox101*Δ (YSB3136), *zfc6*Δ (YSB1954), *apn2*Δ (YSB1430), *fzc36*Δ (YSB2523), *fzc14*Δ (YSB1847), *rim101*Δ (YSB1367), *fzc19*Δ (YSB2116), *fkh2*Δ (YSB1340), *fzc29*Δ (YSB719), *crl6*Δ (YSB1107), *fzc24*Δ (YSB775), *gln3*Δ (YSB3155), *gat203*Δ (YSB570), *asg101*Δ (YSB2698), *yrm103*Δ (YSB2299), *fzc15*Δ (YSB647), *pip201*Δ (YSB3100), *stb4*Δ (YSB1014), *fzc51*Δ (YSB1843), *bzp2*Δ (YSB2703), *zfc4*Δ (YSB2232), *zfc7*Δ (YSB482), *ada2*Δ (YSB2382), *zap103*Δ (YSB2541), *rds2*Δ (YSB1899), *fzc2*Δ (YSB1051), *fzc48*Δ (YSB2647), and *yap1*Δ (YSB1290). (c) set1: *nrg1*Δ (YSB3096), *hob1* Δ (YSB2308), *fzc31*Δ (YSB3093), *pan1*Δ (YSB1181), *met32*Δ (YSB1179), *cuf1*Δ (YSB2665), *gat6*Δ (YSB1384), *hlh5*Δ (YSB2609), *mcm1*Δ (YSB1302), *bzp3*Δ (YSB1099), *aro80*Δ (YSB714), *fzc11*Δ (YSB845), *hlh2*Δ (YSB1147), *asg1*Δ (YSB3013), *hob2*Δ (YSB2282), *clr3*Δ (YSB1834), *mlr1*Δ (YSB2727), *fzc7*Δ (YSB2704), *fzc42*Δ (YSB690), *fzc28*Δ (YSB2337), *gat7*Δ (YSB2699), *fzc8*Δ (YSB2112), *fkh101*Δ (YSB1856), *fzc13*Δ (YSB2517), *fzc3*Δ (YSB2611), *hlh3*Δ (YSB2329), *hcm101*Δ (YSB2390), *yrm101*Δ (YSB2997), *fzc27*Δ (YSB582), *gat1*Δ (YSB2973), *zfc8*Δ (YSB3031), *fzc34*Δ (YSB501), *zfc3*Δ (YSB2108), *fzc32*Δ (YSB2385), *hap1*Δ (YSB2481), *sip4*Δ (YSB2680), *bzp4*Δ (YSB1894), *grf1*Δ (YSB796), *mbs1*Δ (YSB488). set2: *nrg1*Δ (YSB3097), *hob1*Δ (YSB2309), *fzc31*Δ (YSB3094), *pan1*Δ (YSB1183), *met32*Δ (YSB1178), *cuf1*Δ (YSB2666), *gat6*Δ (YSB1386), *hlh5*Δ (YSB3059), *mcm1*Δ (YSB1303), *bzp3*Δ (YSB1100), *aro80*Δ (YSB715), *fzc11*Δ (YSB846), *hlh2*Δ (YSB1149), *asg1*Δ (YSB3014), *hob2*Δ (YSB2283), *clr3*Δ (YSB1836), *mlr1*Δ (YSB2728), *fzc7*Δ (YSB2705), *fzc42*Δ (YSB687), *fzc28*Δ (YSB2338), *gat7*Δ (YSB2700), *fzc8*Δ (YSB2113), *fkh101*Δ (YSB1855), *fzc13*Δ (YSB2518), *fzc3*Δ (YSB2664), *hlh3*Δ (YSB2330), *hcm101*Δ (YSB2391), *yrm101*Δ (YSB2998), *fzc27*Δ (YSB583), *gat1*Δ (YSB2972), *zfc8*Δ (YSB3032), *fzc34*Δ (YSB2979), *zfc3*Δ (YSB2386) *fzc32*Δ (YSB2526), *hap1*Δ (YSB2482), *sip4*Δ (YSB2681), *bzp4*Δ (YSB1895), *grf1*Δ (YSB797), *mbs1*Δ (YSB489). (d) set1: *gat201*Δ (YSB3300), *pdr802*Δ (YSB2387), *gat5*Δ (YSB3033), *ddt1*Δ (YSB1583), *sre1*Δ (YSB2493), *fzc30*Δ (YSB2447), *fzc38*Δ (YSB777), *znf2*Δ (YSB2740), *zfc1*Δ (YSB2573), *fzc40*Δ (YSB3088), *hcm1*Δ (YSB1850), *fzc9*Δ (YSB2984), *fzc44*Δ (YSB2182), *fzc4*Δ (YSB2724), *yap2*Δ (YSB1416), *liv4*Δ (YSB2089),

*fzc23*Δ (YSB3105), *rum1*Δ (YSB3164), *fzc45*Δ (YSB2221), *ccd4*Δ (YSB706), *fzc10*Δ (YSB3083), *fzc26*Δ (YSB3084), *hob7*Δ (YSB3026), *fzc20*Δ (YSB3128), *fzc50*Δ (YSB3131), *miz1*Δ (YSB2133), *ert1*Δ (YSB693), *fzc39*Δ (YSB1820), *clr4*Δ (YSB3282), *hlh4*Δ (YSB2244), and *fap1*Δ (YSB813). set2: *gat201*Δ (YSB3301), *pdr802*Δ (YSB2388), *gat5*Δ (YSB3034), *ddt1*Δ (YSB2633), *sre1*Δ (YSB2494), *fzc30*Δ (YSB2448), *fzc38*Δ (YSB777), *zfc1*Δ (YSB2574), *fzc40*Δ (YSB3088), *hcm1*Δ (YSB1851), *fzc9*Δ (YSB3266), *fzc44*Δ (YSB2181), *fzc4*Δ (YSB2725), *yap2*Δ (YSB1417), *liv4*Δ (YSB2089), *fzc23*Δ (YSB3106), *rum1*Δ (YSB3164), *fzc45*Δ (YSB2222), *ccd4*Δ (YSB707), *fzc10*Δ (YSB3368), *fzc26*Δ (YSB3085), *hob7*Δ (YSB3027), *fzc20*Δ (YSB3129), *fzc50*Δ (YSB3132), *miz1*Δ (YSB3366), *ert1*Δ (YSB694), *fzc39*Δ (YSB2621), *clr4*Δ (YSB3283), *hlh4*Δ (YSB2245), and *fap1*Δ (YSB817)].

Supplementary Table 1. Transcription factors involved in antifungal agent resistance in *C. neoformans*

Antifungal agents	TF mutants showing increased resistance	TF mutants showing increased susceptibility
Azole (Fluconazole)	***HOB1***, *HAP2*, *SKN7*, ***NRG1***, ***MBS1***, *PPR1*, ***JJJ1***, ***HCM1***, *ADA2*, *FZC9*, *GAT7*, ***ERT1***, *FKH2*, ***ECM22***, *DDT1*,*GAT5, YRM103*, ***CUF1***, *FZC51*, *LIV4,*	*BZP3*, *HLH3*, *BZP1*/*HXL1*, ***SRE1***, *RIM101*, *YAP2*, *HLH1*, *YAP4*, *PIP2*, *MIZ1*, *MLN1*, *HOB6*, *MBF1*, *MET32*, *FZC46*, ***YAP1***, *FZC14*, *FZC2, HSF2, ZFC6, FZC45, FZC30, ASG1, STE12, LIV1, FZC22, FZC31, PAN1, BZP2, SP1/CRZ1*, *BZP5*, *SXI1alpha, FZC34, , FZC17, HLH2*
Polyene (Amphotericin B)	***SRE1***, ***YAP1***, *FZC51, SKN7, CLR1, BZP4, ATF1, FZC4*	***HOB1***, ***MBS1***, ***JJJ1***, ***ERT1, ECM22***, *GAT201*, *ZAP104*, *SP1/CRZ1*, *FZC6*, *BZP5*, *HLH1*, *PIP2*, ***HCM1***, *BZP2*, *USV101*, *HOB4*, *STE12*, *HOB5*, *GRF1*, *HEL2*, *FZC45*, *ASG1, FZC22, HOB6, PAN1,***CUF1***, *FZC49, FZC1, BWC2, FAP1, FZC44, FZC8, FZC23, GAT204,* ***NRG1***, *PIP201, RIM101, HLH3, BZP3, MLN1, MET32, ZFC2, FZC31, RUM1, PDR802, FZC10, HLH2*
5-flucyotosine	*HLH3*, *RIM101*, *GAT204*, *HOB3*, *FZC50*, *ZNF2, RDS2, FZC31*	*NRG1*, *ZFC2*, *YAP1*, *MBS1*, *FZC6*, *YAP2*, *BZP3*, *JJJ1*, *HLH1*, *PIP2*, *APN2*, *FZC46*, *HAP2*, *FZC51*, *BZP5, HCM1, FZC19, BZP2, FZC44*
Phenylpyrrole Fungicide (Fludioxonil)	*NRG1*, *JJJ1*, *SP1/CRZ1*, *SKN7*, *GAT7*, *FAP1, ZFC2, GAT204, ZNF2, HEL2, FZC50, SRE1*	*USV101*, *ADA2*, *YAP1*, *FZC6*, *HLH1*, *PIP2*, *FZC46*, *HAP2*, *BZP1*/*HXL1*, *FKH2*, *LIV1*, *YAP2*, *BZP2*, *FZC21*, *HLH3*, *YRM101*, *BZP5*, *GLN3*, *ZFC8*, *DDT1*, *FZC22, HOB6, RLM1, MLN1, PAN1, FZC35, YRM103, ZFC3, ASG1, FZC41, FZC43, FZC51, HAP1, MET32, FZC32*

Supplementary Table 2. Transcription factors involved in the virulence of *C. neoformans*

		Insect host	Murine host	Phenotypes			
CNAG	Gene	RMS [mt/wt]	STM score	Capsule	Melanin	Urease	Stress responses
**01551**	***GAT201***	**1.9***	**-11.125↓↓↓**	↓↓↓	-	↑	Ox/ER/Cm/Hm
**03894**	***PDR802***	**1.73***	**-7.212↓↓↓**	-	-	-	-
**06134**	***BZP1/HXL1***	**>1.67***	**-6.82↓↓↓**	-	-	-	Th/Gx/ER/Cw/Hm
05392	*ZAP104*	1.00	-5.528↓↓	↓↓↓	-	↓↓	-
**04878**	***FZC1***	**1.2***	**-5.365↓↓**	↑↑	↑↑↑	↑	Th/Ox/Gx/Cm
02774	*MAL13*	0.92	-5.219↓↓	-	-	-	-
04583	*DDT1*	1.00	-4.832↓↓↓	-	-	-	Ox/ER
**01431**	***HOB1***	**1.46***	**-4.95↓↓**	-	↓↓↓	-	Th/Os/Ox/Gx/ER/Cm/Cw
**02435**	***BWC2***	**1.5***	**-4.663↓↓**	-	-	-	Ox/ER/Cm
**05420**	***USV101***	**1.5***	**-4.684↓↓**	↑↑	↓↓↓	↑	Th/Ox/ER/Cm
**04804**	***SRE1***	**2.04***	-4.643↓↓	↑↑	-	↓	Th/Ox/Gx/ER/Cm/Hm
00460	*LIV1*	1.00	-3.875↓↓	↓	-	-	Ox
**01973**	***ZFC2***	**1.34***	**-3.554↓↓**	-	-	-	Ox/ER
04841	*FZC43*	1.00	-3.403↓↓	-	-	-	Os
04184	*FZC47*	0.83	-3.37↓↓	↓↓	-	-	Hm
**05222**	***NRG1***	**1.33***	**-3.226↓↓**	↑↑	↑↑	-	Th/Os/Ox/Gx/ER/Cm/Cw
03132	*FZC5*	1.00	-2.832↓	-	-	-	-
00791	*HLH1*	1.00	-2.408↓↓	-	↑↑	↓↓	Ox/ER
01438	*MBS2*	1.00	-2.443↓	-	↓	-	Th/Ox/Hm
07011	*FZC22*	0.83	-2.31↓	-	-	-	Ox/Cm
04353	*CLR1*	0.83	-2.025↓	↑	-	-	Ox/ER/Cm/Hm
04457	*FZC30*	1.00	-1.95↓	↑↑	-	-	Th/Ox/Cm
01173	*PAN1*	1.00	-1.88↓	-	-	-	Os/Ox/ER/Cm
05642	*FZC37*	1.00	-1.813↓	-	-	-	Ox/Hm
05153	*GAT5*	1.00	-1.803↓	-	-	-	Th/Os/Ox/Gx/ER/Cm/Hm
00068	*MET32*	1.00	-1.67↓	-	-	-	Os/Ox/ER
04036	*HSF3*	1.12	-1.65↓	-	-	-	Hm/Cm
**03741**	***FZC31***	**1.17***	**-1.608↓**	-	↑↑	-	Th/Os/Ox/ER/Cm
03336	*FZC50*	1.47*	-0.736	-	-	-	Ox/Cm/Hm
00156	*SP1/CRZ1*	1.37*	-0.485	-	-	-	Th/Ox/ER/Cm/Cw
00239	*YAP1*	1.15*	0.812	↓↓↓	↑↑	↑	Ox/ER/Cm/Hm
04263	*BZP2*	1.58*	1.29	-	-	-	Th/Os/Ox/Gx/ER/Cm/Cw/Hm
00514	*GAT6*	0.99	1.52↓	-	-	-	Th/Ox/Gx/ER/Cm
04012	*FZC18*	1.00	1.59↑	↑	-	-	-
01626	*ADA2*	>1.43*	1.448	↓↓↓	↑↑	-	Th/Os/Ox/Gx/ER/Hm
00841	*FZC40*	1.00	1.688↑	-	-	-	
00830	*FZC38*	1.00	2.172↑	-	-	-	Ox
02603	*ZFC1*	1.70	2.29↑	-	-	-	Cm
03527	*HEL2*	0.83	2.49↑	-	-	-	Os/Ox/ER
05176	*HOB3*	1.00	2.53↑	↓	-	-	ER/Cm
07593	*YAP4*	0.90	2.64↑	-	-	-	ER
06762	*GAT204*	1.50*	2.986↑↑	↓↓	-	-	Ox/Hm
03366	*ZNF2*	1.00	3.351↑↑	-	-	-	Ox/Hm
02322	*FZC17*	0.85	3.443↑↑	↑	-	-	-
07724	*CUF1*	1.20	5.656↑↑	-	↓↓↓	-	Th/Os/Ox/ER/Cm/Cw/Hm

RMS [mt/wt], Relative median survival days = [median survival day for a mutant]/[median survival day for the wt strain (H99); *, *P*<0.05, by Log-rank (Mantel-Cox) test for mutant vs. wt strain. The RMS scores for two-independent strains for each TF mutant were described as an average value.
STM score, Signature tagged mutagenesis based quantitative PCR score = Log2 (output/input) in the lung from the sacrificed mice (average score from three mice). (↓) or (↑) indicates reduced or enhanced virulence, respectively. Average STM scores from two independent mutants for each TF were described.
Capsule/Melanin/Urease, (-) indicates wt-like phenotypes, (↓) or (↑) indicates reduced or enhanced production, respectively. Single arrow (weak changes), Double arrows (moderate changes), Triple arrows (strong changes).
Os, Osmotic stress; Ox, Oxidative stress; Gx, Genotoxic stress; ER, ER-stress; Th, Thermal stress, Cm, Cell membrane stress, Cw, Cell wall stress, Hm, Heavy metal stress. Red and blue color letters indicate enhanced and reduced sensitivity, respectively, to each stress, while black letters indicate that mutants exhibit enhanced or reduced sensitivity to a corresponding stress depending on stress inducing agents.
Bold typed indicate TFs, whose deletions affected virulence at statistically significant levels in both insect and murine models of cryptococcosis.
